# Proteomic analysis unveils host-parasite interactions in *Aedes togoi* infected with *Dirofilaria immitis* and *Brugia pahangi*

**DOI:** 10.1371/journal.pone.0326693

**Published:** 2025-07-09

**Authors:** Wei Yin Vinnie-Siow, Van Lun Low, Hwa Chia Chai, Yvonne Ai-Lian Lim, Tiong Kai Tan

**Affiliations:** 1 Higher Institution Center of Excellence, Tropical Infectious Diseases Research and Education Centre (TIDREC), Universiti Malaya, Kuala Lumpur Malaysia; 2 Department of Biomedical Science, Faculty of Medicine, Universiti Malaya Kuala Lumpur, Malaysia,; 3 Department of Parasitology, Faculty of Medicine, Universiti Malaya Kuala Lumpur, Malaysia; Quinnipiac University, UNITED STATES OF AMERICA

## Abstract

Mosquitoes serve as the primary vectors responsible for transmitting canine filariasis, yet understanding the molecular interactions between filarial parasites and their vectors is a significant challenge.. Therefore, employing a proteomic approach is crucial for elucidating the protein expressions profile in mosquitoes, allowing the tracking of biochemical changes during parasite development and survival within the mosquito. To infer the protein response of mosquitoes to filarial infections, *Aedes togoi* was inoculated with canine filarial parasites, *Dirofilaria immitis* and *Brugia pahangi*, and maintained for 14 days prior before dissection to collect their cuticular tissue proteins for Liquid Chromatography-Tandem Mass Spectrometry (LC-MS/MS) analysis. Actin and prophenoloxidase, recognized as defence proteins, exhibited upregulation in groups inoculated with *D. immitis* and *B. pahangi*. Most proteins in glycolysis, gluconeogenesis, and the TCA cycle were upregulated in both groups, except for dihydrolipoyl dehydrogenase, vital for pyruvate decarboxylation, which was downregulated, while glucose-1-phosphate uridylyltransferase, essential for glycogen production, was expressed despite its absence in the control. Additionally, a pathway related to tyrosine metabolism, involving aspartate aminotransferase, AAEL010442-PA, 4-hydroxyphenylpyruvate dioxygenase, aspartate aminotransferase, and homogentisate 1,2-dioxygenase, was expressed. This study has addressed gaps in understanding the protein response of mosquitoes infected with filarial parasites, shedding light on host defence mechanisms and potential metabolic adaptations, thereby enhancing our comprehension of filariasis infection mechanisms.

## 1. Background

The ability to develop an immune response is one of the most basic evolutionary properties shared by all creatures, including insects. However, the interaction between the filarial parasite and the mosquito vector is a challenging task to investigate. The parasites are successfully adapted if they survive and thrive in the host body. Insects, particularly mosquitoes, possess efficient defence systems to protect themselves against pathogen invasion by producing inducible immune proteins [1]. The defence system of mosquitoes against invading pathogens comprises a complex array of humoral and cellular components. Non-vector refractory mosquitoes demonstrate innate and acquired resistance to filarial infections through mechanisms such as encapsulation or melanisation [2]. However, in the absence of encapsulation or melanisation, microfilariae ingested by the mosquito will develop in the thoracic muscles, progressing from L1 to L2 and ultimately to the L3 infective stage [3]. Hence, to understand the biochemical changes associated with parasite development in the vector, it is crucial to employ a proteomic approach to discern the protein expressions profile in mosquitoes [4].

Proteomic approaches, which include large-scale comprehensive study of the gene expressions at the protein level, could provide direct measurements of protein expression levels and insights into the functions of related proteins [5]. The protein expression profiling of insect immune responses towards pathogens has been reported in several species of insects and their tissue, and many differentially expressed proteins have been identified by mass spectrometry, for example attacin-like proteins, which belongs to the group of antimicrobial peptides were found to be significantly stimulated by challenge with *Escherichia coli* in *Diatraea saccharalis* [6], while phenoloxidase, which involved in melanisation of pathogens and parasites, and two chitinase-like proteins similar to imaginal disc growth factor were found to be elevated following bacterial infection (i.e., *Escherichia coli* and *Micrococcus luteus*) in *Anopheles gambiae* [7]. In addition, the deletion of D7 salivary proteins in *Aedes aegypti* infected with *Plasmodium gallinaceum* showed fewer midgut oocysts, suggesting a hostile miduge environment in the absence of D7 proteins [8].

Various studies have been carried out mainly on the development of tools for the genetic alteration of mosquito vectors, with the ultimate goal of interfering with the life cycle of parasites within the vectors, thereby stopping transmission [9,10]. It is crucial to understand the interaction between insect organs, tissues, and hemolymph, where specific interactions occur, such as the development of larvae in various locations and the biochemical response of mosquitoes to filarial infections [11]. Nevertheless, efforts to discover the protein products that are responsible for the susceptibility to *Dirofilaria* and *Brugia* infections or immune responses to these infections in mosquitoes have not been clearly addressed. As an initial step towards understanding the protein characterization in mosquito immune response and the development of filarial worms, this study aims to compare the protein profiles of *Aedes togoi* infected with *Dirofilaria immitis* and *Brugia pahangi* with those of non-infected mosquitoes.

## 2. Methods

### 2.1. Ethics statement

This study was approved by the Medical Ethics Committee of Department Veterinary Services Malaysia [JPV: BPI/500–4/1/2 (18)] and the Institutional Biosafety and Biosecurity Committee, Universiti Malaya [UMIBBC/NOI/R/TNC/TIDREC-014/13,062,019]. Written informed consents were obtained from the owners of the animal shelters.

### 2.2. Mosquito colonisation

A laboratory strain of *Ae. togoi* was reared and maintained in an insectarium at the Department of Parasitology, Faculty of Medicine, Universiti Malaya, under standard conditions, including a temperature of 26 ± 2°C and a relative humidity (RH) of 80 ± 10%, with a light:dark cycle of L12:D12 hours. In addition, 10% of sugar solution was provided daily to the mosquitoes as food source. Female mosquitoes aged 5–7 days were chosen for the infection experiments.

#### 2.3. Source of microfilariae

Microfilariaemic blood samples (*D. immitis* and *B. pahangi*) were collected from infected dogs in an animal shelter located in Semenyih, Kuala Lumpur, Malaysia. Details on blood collection were published elsewhere [12,13]. Briefly, dog blood samples were collected randomly from the animal shelters and screened for filarial parasites and other pathogens using serological tests and microscopic Giemsa stain techniques. Further species identification of filarioid parasites was performed using molecular techniques. After examination, a one-time blood collection was performed on two dogs: one was positive for a monoinfection of *B. pahangi* and the other with *D. immitis*. Ten milliliters of blood samples were collected in anticoagulated blood tubes containing approximately 1.5 ml of Citrate Phosphate Dextrose Adenine (CDPA) by a trained veterinarian and stored at 4°C until further processing. No medications were given to these animals prior to blood collection. The presence, viability and number of microfilariae (mfs) in all samples obtained were assessed. Approximately 100µl of blood was placed on a glass slide, covered with a coverslip, and microfilariae were counted by examination with a microscope under 10x magnification. The procedure was repeated three times, and an average was taken to determine the total number of microfilariae per ml. Additionally, dog blood samples that tested negative across all types of examination were collected and utilized as a control (control blood). Subsequently, the blood was administered to the mosquitoes using the Hemotek™ system.

#### 2.4. Infection of mosquitoes with microfilariae

The methods for the infection study were previously published in Vinnie-Siow et al. [14]. *Aedes togoi* females were starved overnight before the infection experiments. Three study groups were included: *D. immitis*-inoculated group (DIM), *B. pahangi*-inoculated group (BPH) and negative control. The number of mosquitoes were recorded and separated into several cups and placed inside a glove box approximately 127 cm x 254 cm x 95 cm in dimensions. Mosquitoes were allowed to feed through Parafilm® membrane on blood containing monoinfection of *D. immitis* (1000mf/ml) or *B. pahangi* (9000mf/ml) microfilariae and negative control blood in the Hemotek™ system (Discovery Workshop, UK) having a surface area of 9.62 cm^2^ in the Arthropod Containment Level 2 (ACL2) Laboratory located in the Department of Parasitology, Faculty of Medicine, Universiti Malaya.

Briefly, the temperature of the blood meal was maintained at 37°C using an electric heating element in the Hemotek™ system throughout the blood feeding process. The blood containing either *D. immitis* or *B. pahangi* was administered to the mosquitoes by placing the cups underneath the feeder, ensuring that the cup’s nylon netting was in contact with the feeder’s membrane, and the mosquitoes were fed for 2 hours. Mosquitoes fed with either *D. immitis* or *B. pahangi* infected blood were examined under a stereomicroscope to assess engorgement. Fully engorged females were segregated into new cups, while mosquitoes that did not fully feed or did not feed at all were discarded. To facilitate larval development, mosquitoes were maintained for a period of 14 days [15], after which all mosquitoes were individually dissected. All cuticular materials (entire content of the dissected mosquitoes including all tissues and structures of mosquitoes) were then transferred to sterilized microcentrifuge tubes and stored in Phosphate-Buffered Saline (PBS) at −80°C.

#### 2.5. Protein sample preparation

The cuticular material of each sample with similar filarial infections were pooled (DIM = 9 mosquitoes; BPH = 8 mosquitoes). In addition, nine individuals of clean *Ae. togoi* were used as control group in the current study. The pooled cuticular material was pelleted by centrifugation at 14,800 rpm for 10 minutes at 4°C. Subsequently, the supernatant was removed by micropipette and pelleted cuticular material remained in the tube. Then, drops of liquid nitrogen were added to the sample tube. The samples were homogenised by using the polypropylene disposable pestle. After sample homogenisation was completed, sample tubes were sealed with Parafilm and stored at −80°C prior to extraction.

Eight (8) M urea lysis buffer was added to the homogenized sample at −20°C for protein extraction. Then, the tissue debris of the mosquito was removed by centrifugation at 12,000 rpm for 10 minutes at 4°C. Protein in the supernatant was transferred to a new 1.5 ml microcentrifuge tube. Then, the protein concentration was determined by using Pierce™ BCA Protein Assay Kit (Thermo Fisher Scientific, United States) according to the manufacturer’s protocol.

Protein samples were concentrated using cold acetone. Briefly, four times the total sample volumes of cold acetone were added into the tube. Subsequently, the tubes were vortexed and incubated for 60 minutes at −20°C. After that, the samples were centrifuged at 10,000–11,000 rpm for 10 minutes at 4°C. The supernatant was decanted and the remaining acetone in the tubes were allowed to evaporate at room temperature.

The protein pellets were dissolved in 2M urea aqueous solution and were denatured with 10mM dithiothreitol (DTT) at 56°C for 1 hour followed by alkylation with 50mM iodoacetamide (IAA). Then, the samples were incubated for 1 hour at room temperature in the dark. Afterwards, 500mM ammonium bicarbonate was added into the solution to make a final concentration of 50mM ammonium bicarbonate with pH 7.8. Trypsin (Promega, Wisconsin, United States) was added into the protein solution for digestion at 37°C for 15 hours. Next, the generated peptides were further purified and desalted using C18 SPE column (Thermo Scientific). Finally, the extracted peptides were lypholised to near dryness. The peptides were resuspended in 20μl of 0.1% formic acid before LC-MS/MS analysis.

#### 2.6. Nano LC-MS/MS analysis and database search

Briefly, 1 μg of protein samples was injected into the Ultimate 3000 nano UHPLC system (Thermo Scientific, Waltham, MA). The full scan was performed between 300−1,650 m/z at the resolution of 60,000 at 200 m/z, the automatic gain control target for the full scan was set to 3e6. The MS/MS scan was operated in Top 20 mode using the following settings: resolution 15,000 at 200m/z; automatic gain control target 1e5; maximum injection time 19ms; normalized collision energy at 28%; isolation window of 1.4 Th; charge state exclusion: unassigned, 1, > 6; dynamic exclusion of 30 seconds.

Next, the raw MS/MS data obtained were searched against UniProt database using Maxquant (1.6.2.14) for protein identification. Since *Ae. togoi* database was not fully established (30 sequences in UniProt database), the raw MS/MS data were also searched against *Ae. aegypti* database. The search parameters were set as follows: the protein modifications were carbamidomethylation (C) (fixed) and oxidation (M) (variable); the enzyme specificity was set as trypsin; the maximum missed cleavages were set as 2; the precursor ion mass tolerance was set as 10 ppm, and MS/MS tolerance was 0.5 Da. The protein IDs with the highest scores were selected.

#### 2.7. Data analyses

The protein IDs and intensities from all groups were assembled and analysed using MySQL Workbench. The intensity of the proteins was compared between the groups to determine the upregulations and downregulations of the protein by constructing a heatmap for all expressed proteins in the three study groups in log2 scale. In addition, log2fold changes of the proteins were calculated by using Microsoft Excel 2017, where proteins with [log2(Fold Change) >1.5] was regarded as significant upregulation and those with [log2(Fold Change) <−1.5] was regarded as significant downregulation. Modulated metabolic pathways were generated using UMLetino Version 15.0.0 aided by the Kyoto Encyclopedia of Genes and Genomes (KEGG) database. Cytoscape Version 3.9.0, with the ClueGo pathway enrichment analysis plugin, was used to categorise and integrate the metabolic pathways of all groups (BPH, DIM and Control groups).

## 3. Results

### 3.1. Effects of *B. pahangi* and *D. immitis* challenge on protein expression in *Ae. togoi*

In the present study, the protein expression profiles of filarial-infected (*B. pahangi* and *D. immitis*) *Ae. togoi* and non-infected *Ae. togoi* (control) were compared. The LC-MS/MS analysis revealed a total of 290 proteins from all three groups [i.e., *B. pahangi*-inoculated (BPH), *D. immitis-*inoculated (DIM) and control]. Of these, 192 proteins were observed in all the three group. Sixty-nine proteins were expressed in both DIM and BPH, but were not detected in the control. Meanwhile, one protein was found only in DIM and control, and five proteins were found in both BPH and control.

Ten uniquely expressed proteins were found exclusively in DIM, while 4 and 9 proteins were observed only in BPH and control groups, respectively. A comparison of all proteins expressed in the three groups revealed a total of 104 upregulated proteins (Table S1) and 52 downregulated proteins (Table S2) in both BPH and DIM compared to the control.

A total of 35 proteins exhibited upregulation in DIM but downregulation in BPH, while three proteins (phosphoglucomutase 1, ATP synthase subunit D, mitochondrial, and muscle-specific actin 3) displayed upregulation in BPH but downregulation in DIM (Table S3). Additionally, 69 proteins were identified in both DIM and BPH but were not observed in the control, including acetyl-CoA (acetylcoenayme A) carboxylase, glucose-1-phosphate uridylyltransferase, aspartate aminotransferase, fumarate hydratase, and coronin (Table S4). Moreover, ten proteins, such as homogentisate 1,2-dioxygenase, vitellogenic carboxypeptidase, 4-hydroxyphenylpyruvate dioxygenase, S-methyl-5-thioadenosine phosphorylase, and others (Table S5), were exclusively found in DIM, while four proteins, including pyruvate dehydrogenase E1 component subunit alpha (Fragment), cytochrome C oxidase assembly factor 3, and others (Table S6), were exclusively found in BPH. Furthermore, nine proteins were detected solely in the control group, namely calcium-transporting ATPase, 40S ribosomal protein S16, and various others (Table S7).

To further define the effect of filarial parasites infection on mosquito cellular and physiological responses, a deeper analysis was performed to identify the significantly expressed proteins. The log2 fold Change and t-test were performed, which indicated a total of 34 significantly upregulated proteins found in BPH and DIM (log2 fold change ranging from 1.50 to 5.00 [log_2_(fold change)>1.5 and p-value <0.05], compared to control (Table S1). For downregulated proteins [log_2_(fold change) <−1.5 and p-value <0.05], 13 proteins (log2fold change ranging from −1.50 to −7.98) in BPH and DIM were significantly expressed (Table S2). DIM exhibited a higher response compared to BPH in terms of upregulation. The proteins exhibiting the highest enrichment for DIM and BPH were fructose-bisphosphatase (BPH: log2 fold change: 3.66, fold change: 12.60-folds; DIM: log2 fold change: 5.0, fold change: 32.0-folds) and phosphofructo-2-kinase (BPH: log2 fold change: 4.13, fold change: 17.50-folds; DIM: log2 fold change: 4.95, fold change: 30.94-folds). Conversely, the PKS_ER domain-containing protein (BPH: log2 fold change: −7.98, fold change: −253.17 folds, DIM: log2 fold change: −5.45, fold change: −43.70-folds) and tropomyosin invertebrate (BPH: log2 fold change: −4.86; fold change: −28.37-folds; DIM: log2fold change: −4.03; fold change: −16.36-folds) were the most downregulated proteins.

Only a few proteins were significantly expressed with inconsistent upregulation and downregulation in BPH (significant upregulation: 1 out of 35, 2.86%; significant downregulation: 2 out of 35, 5.71%) and DIM (significant upregulation: 8 out of 35, 22.86%) (Table S3). In BPH, muscle-specific actin 3 was significantly upregulated and Vitellogenin-B (fold change: 4.17; log2fold change: −2.06) and proteasome subunit alpha type (fold change: 4.96; log2fold change: −2.31) were significantly downregulated. In contrast, glucose-6-phosphate isomerase (fold change: 4.75; log2 fold change: 2.25), succinate--CoA ligase ADP-forming subunit beta, mitochondrial (fold change: 5.30; log2 fold change: 2.41), delta-1-pyrroline-5-carboxylate synthase (fold change: 3.36; log2 fold change: 1.750), AAEL010097-PA (fold change: 3.27; log2 fold change: 1.71) vitellogenin-B (fold change: 8.97; log2fold change: 3.17), triosephosphate isomerase (fold change: 3.71; log2 fold change: 1.89), 40S ribosomal protein S3 (fold change: 4.23; log2 fold change: 2.08) and delta-1-pyrroline-5-carboxylate synthase (fold change: 3.36; log2 fold change: 1.75) were significantly upregulated in DIM.

Interestingly, the multifunctional fusion protein was absent in BPH, but it was downregulated (log2 fold change: −0.68, fold change: 1.60) in the DIM group (Table S8). On the other hand, five proteins (Table S9) found in BPH were not detected in DIM. Out of the five proteins, three proteins [i.e., AAEL008289-PA (log2 fold change: 0.68, fold change: 1.60), NADH (Nicotinamide adenine dinucleotide hydrogen) dehydrogenase [ubiquinone] 1 alpha subcomplex subunit 10 (log2 fold change: 0.23, fold change: 1.17) and laminin gamma 1 chain (log2 fold change: 1.14, fold change: 2.20)] were upregulated and two proteins [i.e., AAEL003110-PA (log2 fold change: −1.03; fold change: −2.04) and alpha-galactosidase (log2 fold change: −1.03, fold change: −2.04)] were downregulated. However, none of these five proteins were significantly expressed. Additionally, nine proteins were uniquely found in the control group. These included calcium-transporting ATPase, 40S ribosomal protein S16, AAEL015512-PA, AAEL007494-PA, AAEL003957-PA, AAEL006014-PA, AAEL003658-PA, AAEL012905-PA and AAEL002759-PB.

Some proteins known to be involved in immune regulation of mosquitoes were identified after a literature review (Tables S1 and S2) [i.e., actin was significantly upregulated in both DIM (log2 fold change: 2.55, fold change: 5.84) and BPH (log2 fold change: 2.34; fold change: 5.07); prophenoloxidase was significantly upregulated in DIM (log2fold change: 1.60; fold change: 3.04) but not significantly upregulated in BPH (log2 fold change: 1.10, fold change: 2.14); In addition, insignificant downregulations of arginine kinase and transferrin were observed in both study groups.

#### 3.2. Effects of *D. immitis* and *B. pahangi* challenge on metabolic changes in *Ae. togoi*

**Fig 1:**
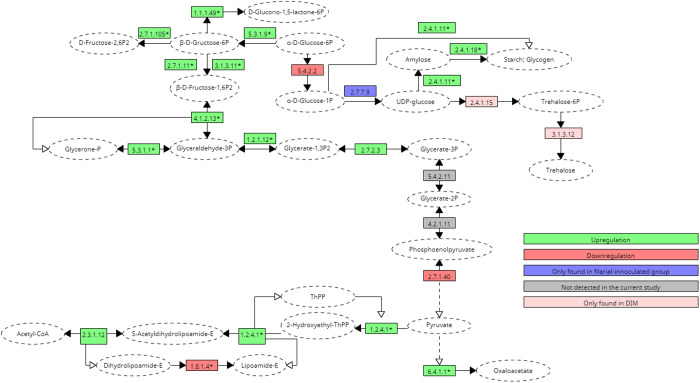
Postulated carbohydrate metabolism pathway of the proteins upregulated in DIM.

**Fig 2:**
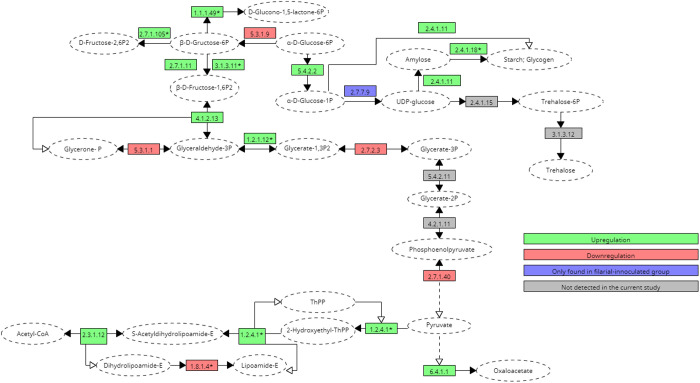
Postulated carbohydrate metabolism pathway of the proteins upregulated in BPH.

The overall enzymes/proteins exhibited upregulation in glycolysis, gluconeogenesis, and the TCA cycle, as depicted in Figs 1 and 2. The glycolysis- tricarboxylic acid (glycolysis-TCA) cycle is a significant metabolic pathway that plays an essential role in energy-processing reactions and serves as a source of metabolites for other biological events. To discover the orthologous links of key metabolic enzymes, the glycolysis/gluconeogenesis pathways and tricarboxylic acid (TCA) cycle were evaluated in the current study. In general, a significant degree of similarity was discovered among the three groups (DIM, BPH and control) to anticipate orthology in most cases and to determine the upregulation and downregulation of the specific enzymes or proteins in *Ae. togoi* after the filarial challenge (*D. immitis* and *B. pahangi*).

Variations in the number of enzymatic activities in one or more groups were detected, and these changes appear to be more prevalent in enzymes involved in glycolysis-TCA cycle and gluconeogenesis. The enzymes and proteins involved in glycolysis and gluconeogenesis found in all studied groups were glyceraldehyde-3-phosphate dehydrogenase (1.2.1.12), ATP-dependent-6-phosphofructokinase, fructose-bisphosphatase (2.7.1.11), triosephosphate isomerase (5.3.1.1), phosphofructo-2-kinase (2.7.1.105), pyruvate dehydrogenase E1 component subunit beta (1.2.4.1), phosphoglycerate kinase (2.7.2.3), pyruvate kinase (2.7.1.40), phosphoglucomutase (5.4.2.2), glucose-6-phosphate isomerase (5.3.1.9), glucose-6-phosphate-1-dehydrogenase (1.1.1.49), glucose-1-phosphate uridylyltransferase (2.7.7.9), glycogen starch synthase (2.4.1.11) and 1,4-alpha-glucan-branching enzyme (2.4.1.18) (Table 1).

**Table 1:** Proteins found in DIM and BPH involved in the metabolic pathway and their accession ID as demonstrated in Figs 1 and 2.

No	ID number	Pathway involved	Protein	Protein IDs
1.	6.4.1.1	TCA	Pyruvate carboxylase	Q16V52;A0A6I8TI71;Q16921
2.	2.3.1.12	TCA	Dihydrolipoyllysine-residue succinyltransferase component of 2-oxoglutarate dehydrogenase complex, mitochondrial	Q17H89;A0A6I8T705
3.	1.2.1.12	Glycolysis and gluconeogenesis	Glyceraldehyde-3-phosphate dehydrogenase	J9HYM2;A0A292AH36
4.	2.7.1.11	Glycolysis and gluconeogenesis	ATP-dependent 6-phosphofructokinase	Q174J0;A0A6I8TDP2;A0A6I8TDB1
5.	4.1.2.13	Glycolysis and gluconeogenesis	Fructose-bisphosphate aldolase	Q178U9;Q178U8
6.	3.1.3.11	Glycolysis and gluconeogenesis	Fructose-bisphosphatase	Q17M22;Q17M23
7.	5.3.1.1	Glycolysis and gluconeogenesis	Triosephosphate isomerase	Q17HW3
8.	2.7.1.105	Glycolysis and gluconeogenesis	Phosphofructo-2-kinase	A0A6I8TF90;A0A6I8TNE7;A0A6I8TNB3;Q16K25;Q16K24;Q16K23;A0A6I8TMK5;A0A6I8TPQ4;A0A6I8TF87
9.	1.2.4.1	TCA	Pyruvate dehydrogenase E1 component subunit beta	Q17D51;A0A1S4F7M2
10.	6.4.1.2	Pyruvate metabolism	Acetyl-CoA carboxylase	A0A6I8U7E0;A0A6I8U419;A0A6I8TTM8;A0A6I8U6N7;A0A6I8U908;Q176P0
11.	2.7.2.3	Glycolysis and gluconeogenesis	Phosphoglycerate kinase	Q95UR6;Q8WQL0;Q8WQK9;Q95UR5;Q8WQK8;Q8WQL1;B1A651
12.	2.7.1.40	Glycolysis and gluconeogenesis	Pyruvate kinase	Q16LP4;Q16LP5;Q16F38
13.	5.4.2.2	Glycolysis and gluconeogenesis	Phosphoglucomutase	Q58I84;Q16U43
14.	5.3.1.9	Glycolysis and gluconeogenesis	Glucose-6-phosphate isomerase	Q16LE8;Q16KI0
15.	1.1.1.49	Glycolysis and gluconeogenesis	Glucose-6-phosphate 1-dehydrogenase	A0A6I8THL2;Q0IEL8
16.	2.7.7.9	Glycolysis and gluconeogenesis	Glucose-1-phosphate uridylyltransferase	A0A6I8TBP2;Q58I82;A0A1S4FB16
17.	2.4.1.11	Glycolysis and gluconeogenesis	Glycogen starch synthase	Q17DG0
18.	2.4.1.15	Glycolysis and gluconeogenesis	Trehalose −6-phosphate phosphatase	Q16S69;A0A1S4FR40
19.	2.4.1.18	Glycolysis and gluconeogenesis	1,4-alpha-glucan-branching enzyme	Q16SE5;Q16PC7
20.	3.1.3.12	Glycolysis and gluconeogenesis	Trehalose −6-phosphate phosphatase	Q16S69;A0A1S4FR40
21.	1.8.1.4	TCA cycle	Dihydrolipoyl dehydrogenase	Q174D6;A0A1S4FF23

Additionally, trehalose-6-phosphatase (2.4.1.15; 3.1.3.12) which removes phosphate from trehalose 6-phosphate (Tre6P) to produce free trehalose and also catalyses the dephosphorylation of glucose- 6- phosphate (Glu6P) and 2-deoxyglucose-6-phosphate was found exclusively in the DIM group (Table 1). This enzyme is involved in glycolysis and gluconeogenesis pathways. Moreover, pyruvate carboxylase (6.4.1.1), dihydrolipoyllysine-residue succinyltransferase component of 2-oxoglutarate dehydrogenase complex, mitochondrial (2.3.1.12) which are involved in the TCA cycle were also identified in current study. On the other hand, acetyl-CoA carboxylase (6.4.1.2) involved in the pyruvate metabolism was also seen in the study. However, two crucial proteins for glycolysis and gluconeogenesis were not found in the current study: 2,3-bisphosphoglycerate-dependent phosphoglycerate mutase (5.4.2.11) which catalyses the interconversion of 2-phosphoglycerate and 3-phosphoglycerate; and phosphopyruvate hydratase (4.2.1.11), a hydro-lyase that catalyses the dehydration of 2-phosphoglycerate to produce phosphoenolpyruvate (Table 2).

**Table 2:** Other proteins involved in the metabolic pathway demonstrated in Figs 1 and 2.

No	ID number	Pathway involved	Protein
1	5.4.2.11	Glycolysis and gluconeogenesis	2,3-bisphosphoglycerate-dependent phosphoglycerate mutase
2	4.2.1.11	Glycolysis and gluconeogenesis	Phosphopyruvate hydratase

Most enzymes/proteins involved in glycolysis, gluconeogenesis, and the TCA cycle showed significant upregulation in both DIM and BPH (Figs 1 and 2). However, dihydrolipoyl dehydrogenase (or dihydrolipoamide acetyltransferase) (DIM: log2 fold change: −2.42, fold change: −5.36; BPH: log2 fold change: −2.91, fold change: −7.51), responsible for pyruvate decarboxylation and vital for energy production in cells involved in the TCA cycle, was observed to be downregulated in both DIM and BPH. Additionally, glucose-1-phosphate uridylyltransferase, which catalyses the conversion of glucose-1-phosphate into UDP-glucose, a crucial precursor for glycogen production, was absent in the control but expressed in both DIM and BPH.

Upon comparison between the metabolic pathways of DIM and BPH, a few proteins showed inconsistent upregulation and downregulation between both groups. Triosephosphate isomerase (DIM = log2 fold change: 1.89, fold change: 3.71, BPH = log2fold change: −0.13, fold change: −1.10), phosphoglycerate kinase (DIM = log2 fold change: 0.57, fold change: 1.48, BPH = log2 fold change: −1.07, fold change: −2.10) and glucose-6-phosphate isomerase (DIM = log2 fold change: 2.25, fold change: 4.75, BPH = log2 fold change: −0.75, fold change: −1.68), pyruvate kinase (DIM: log2 fold change: 0.54, fold change: 1.45; BPH: log2 fold change: −1.21; fold change: −2.32) were upregulated in DIM but downregulated in BPH. On the other hand, phosphoglucomutase (DIM = log2 fold change: −0.56, fold change: −1.48, BPH = log2 fold change: 0.47, fold change: 1.39) that catalyses the interconversion of glucose-6-phosphate (G-6-P) and glucose-1-phosphate was downregulated in DIM but upregulated in BPH

### 3.3. Functional enrichment analysis

**Fig 3:**
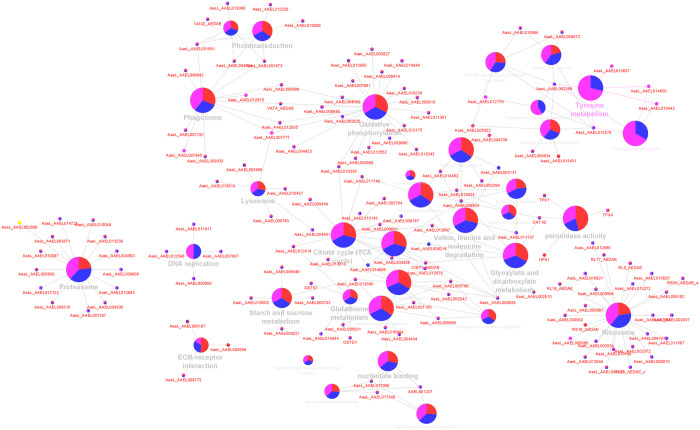
ClueGO enrichment analysis of Gene Ontology terms and KEGG for BPH and DIM and control. Nodes represent significantly enriched terms (Bonferroni step-down corrected p < 0.05) and are cluster according to their groups (BPH, DIM and control).

**Fig 4:**
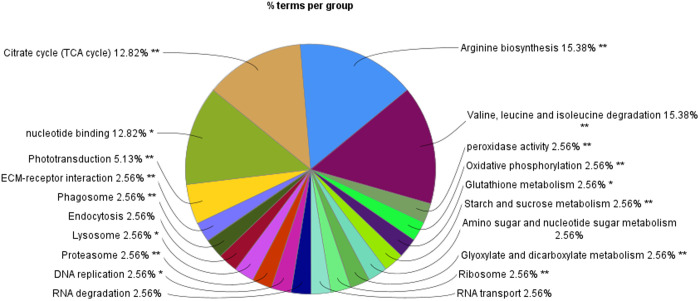
Functional analysis of KEGGs and GO terms for all proteins expressed in the three groups (BPH, DIM and control) identified by LC-MS/MS on Cytoscape using the plug-in ClueGO.

**Fig 5:**
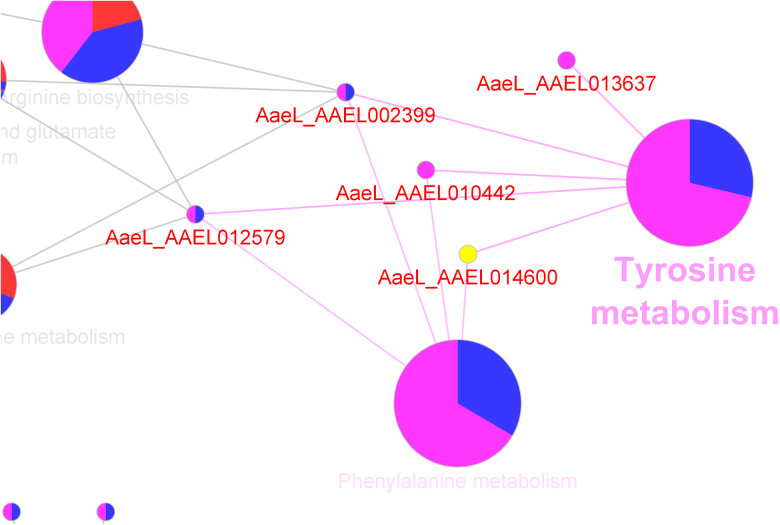
Unique KEGG pathways which were only expressed in DIM and BPH.

Functional enrichment terms were applied to the GO annotations to categorise and integrate into the metabolic pathways using ClueGO pathway enrichment analysis (Fig 3, Tables 3 and 4). Based on the coordinated expression of proteins enriching the KEGG pathways and Gene Ontology Biological Process terms, the significance of associated functional pathways expressed in *Ae. togoi* after infection with *B. pahangi* and *D. immitis* were predicted by the software. The major biological pathways identified using ClueGO pathway enrichment analysis (Fig 4) were associated with arginine biosynthesis (15.38%), valine, leucine and isoleucine degradation (15.38%), citrate cycle-TCA cycle (12.82%), nucleotide binding (12.82%) and phototransduction (5.13%). Other associated biological pathways were also found in the analysis which included ECM-receptor interaction, phagosome, endocytosis, lysosome, proteasome, DNA replication, RNA degradation, peroxidase activity, oxidative phosphorylation, glutathione metabolism, starch and sucrose metabolism, amino sugar and nucleotide sugar metabolism, glyoxylate and dicarboxylate metabolism, ribosome and RNA transport each with 2.56%. Although most of the metabolic pathways were not altered due to the response to BPH and DIM, a pathway related to tyrosine metabolism was significantly expressed (Bonferroni-corrected p values <0.01) in BPH and DIM (Fig 5 and Table 5).

**Table 3:** List of enriched Gene ontology (GO) terms pathway for all groups extracted from ClueGO cytoscape analysis. The number of associated proteins represented using gene name in each pathway with their corresponding term p-value are shown.

Term	P Value	Associated Genes (%)	No. Genes	% Genes			Associated gene found
				Control	BPH	DIM	
Peroxidase activity	0.0001599048	40.00	4	45.77	21.76	32.47	[CAT1B, TPX1, TPX4]
Organonitrogen compound metabolic process	0.0635897815	5.49	5	29.67	35.26	35.07	[AaeL_AAEL016984, AaeL_AAEL017096, AaeL_AAEL017451, GSTD1, SRPN9]
Protein metabolic process	0.0560822226	4.48	3	26.01	37.09	36.90	[AaeL_AAEL017096, AaeL_AAEL017451, SRPN9]
Nucleotide binding	0.0020844080	21.05	4	28.34	35.92	35.74	[AAEL801237, AaeL_AAEL016984, AaeL_AAEL017096, AaeL_AAEL017349]
Purine ribonucleotide binding	0.0119680641	18.75	3	26.01	37.09	36.90	[AAEL801237, AaeL_AAEL017096, AaeL_AAEL017349]
Nucleoside-triphosphatase activity	0.0026894170	33.33	3	26.01	37.09	36.90	[AAEL801237, AaeL_AAEL017096, AaeL_AAEL017349]

**Table 4:** List of enriched KEGG pathways for all groups extracted from ClueGO cytoscape analysis. The number of associated proteins represented using gene name in each pathway with their corresponding term p-value are shown.

Term	P Value	Associated Genes (%)	No. Genes	% Genes			Associated gene found
				control	BPH	DIM	
Oxidative phosphorylation	0.0000000000	15.49	22	32.30	33.94	33.76	[AaeL_AAEL002825, AaeL_AAEL002827, AaeL_AAEL004060, AaeL_AAEL004423, AaeL_AAEL005508, AaeL_AAEL005610, AaeL_AAEL007681, AaeL_AAEL007777, AaeL_AAEL008848, AaeL_AAEL009066, AaeL_AAEL009414, AaeL_AAEL009808, AaeL_AAEL010230, AaeL_AAEL010330, AaeL_AAEL011381, AaeL_AAEL012035, AaeL_AAEL012175, AaeL_AAEL012552, AaeL_AAEL013043, AaeL_AAEL014944, VATA_AEDAE]
RNA transport	0.0757019743	4.11	6	30.53	34.83	34.64	[AaeL_AAEL010318, AaeL_AAEL013359, AaeL_AAEL017096, EIF3A_AEDAE, EIF3B_AEDAE, EIF3F_AEDAE]
RNA degradation	0.0562225431	6.56	4	34.52	32.82	32.65	[AaeL_AAEL006895, AaeL_AAEL008500, AaeL_AAEL010318, AaeL_AAEL011584]
DNA replication	0.0148085784	11.11	4	0.00	50.13	49.87	[AaeL_AAEL000999, AaeL_AAEL007007, AaeL_AAEL011811, AaeL_AAEL012546]
Proteasome	0.0000000000	35.90	14	23.15	38.52	38.32	[AaeL_AAEL002906, AaeL_AAEL003871, AaeL_AAEL004308, AaeL_AAEL004563, AaeL_AAEL005830, AaeL_AAEL006315, AaeL_AAEL007297, AaeL_AAEL009898, AaeL_AAEL010048, AaeL_AAEL010087, AaeL_AAEL012122, AaeL_AAEL012943, AaeL_AAEL013236, AaeL_AAEL014723]
Lysosome	0.0128498338	7.50	6	29.67	35.26	35.07	[AaeL_AAEL004931, AaeL_AAEL005763, AaeL_AAEL007777, AaeL_AAEL010437, AaeL_AAEL013614]
Endocytosis	0.0564397201	4.13	5	26.02	32.98	41.01	[AaeL_AAEL003503, AaeL_AAEL007845, AaeL_AAEL009317, AaeL_AAEL010403, AaeL_AAEL013614]
Phagosome	0.0000000166	17.14	12	31.14	29.60	39.26	[AaeL_AAEL001673, AaeL_AAEL001951, AaeL_AAEL003503, AaeL_AAEL004631, AaeL_AAEL006642, AaeL_AAEL007132, AaeL_AAEL007777, AaeL_AAEL007845, AaeL_AAEL009808, AaeL_AAEL012035, AaeL_AAEL012875, VATA_AEDAE]
ECM-receptor interaction	0.0064728856	25.00	3	51.31	32.52	16.17	[AaeL_AAEL008773]
Glutathione metabolism	0.0125885792	9.43	5	29.67	35.26	35.07	[AaeL_AAEL005931, AaeL_AAEL010644, AaeL_AAEL012614, GSTD1, GSTS1]
Starch and sucrose metabolism	0.0013792341	16.13	5	34.52	32.82	32.65	[AaeL_AAEL000703, AaeL_AAEL004221, AaeL_AAEL006446, AaeL_AAEL010602, AaeL_AAEL012665]
Amino sugar and nucleotide sugar metabolism	0.0793164074	6.00	3	14.95	42.64	42.41	[AaeL_AAEL002781, AaeL_AAEL004931, AaeL_AAEL012665]
Glyoxylate and dicarboxylate metabolism	0.0000056542	23.33	7	32.73	36.31	30.96	[AaeL_AAEL002510, AaeL_AAEL006634, AaeL_AAEL006928, AaeL_AAEL012897, CAT1B, CISY1_AEDAE]
Ribosome	0.0000000000	15.92	25	22.58	37.99	39.43	[AaeL_AAEL000010, AaeL_AAEL000987, AaeL_AAEL002047, AaeL_AAEL002372, AaeL_AAEL002534, AaeL_AAEL003582, AaeL_AAEL005085, AaeL_AAEL008103, AaeL_AAEL008192, AaeL_AAEL009747, AaeL_AAEL009994, AaeL_AAEL010168, AaeL_AAEL010821, AaeL_AAEL011587, AaeL_AAEL012585, AaeL_AAEL012686, AaeL_AAEL012944, AaeL_AAEL013272, AaeL_AAEL013625, RL17_AEDAE, RL18_AEDAE, RL23_AEDAE_c, RL5_AEDAE, RS3A_AEDAE_a]
Hippo signaling pathway	0.0137392478	8.47	5	29.67	35.26	35.07	[1433Z_AEDAE, AaeL_AAEL001673, AaeL_AAEL001951, AaeL_AAEL004631, AaeL_AAEL013068]
Phototransduction	0.0021818590	14.29	5	34.52	32.82	32.65	[AaeL_AAEL001673, AaeL_AAEL001951, AaeL_AAEL004631, AaeL_AAEL010506, AaeL_AAEL012326]
Glycolysis/ gluconeogenesis	0.0000000011	25.00	11	33.43	34.96	31.61	[AaeL_AAEL001158, AaeL_AAEL002542, AaeL_AAEL004338, AaeL_AAEL005766, AaeL_AAEL006895, AaeL_AAEL006928, AaeL_AAEL012576, AaeL_AAEL012665, AaeL_AAEL013613, AaeL_AAEL016984]
Citrate cycle (TCA cycle)	0.0000000000	44.12	15	29.29	37.97	32.74	[AaeL_AAEL000454, AaeL_AAEL002764, AaeL_AAEL004338, AaeL_AAEL006928, AaeL_AAEL008167, AaeL_AAEL009691, AaeL_AAEL010143, AaeL_AAEL010330, AaeL_AAEL011746, AaeL_AAEL012614, AaeL_AAEL012897, AaeL_AAEL013613, CISY1_AEDAE]
Pentose phosphate pathway	0.0000075099	30.00	6	34.52	32.82	32.65	[AaeL_AAEL001158, AaeL_AAEL004434, AaeL_AAEL005766, AaeL_AAEL005931, AaeL_AAEL006895, AaeL_AAEL012665]
Fructose and mannose metabolism	0.0134751918	12.12	4	34.52	32.82	32.65	[AaeL_AAEL001158, AaeL_AAEL002542, AaeL_AAEL005766, AaeL_AAEL006895]
Pyruvate metabolism	0.0000015782	21.62	8	29.66	37.60	32.73	[AaeL_AAEL004338, AaeL_AAEL006634, AaeL_AAEL006928, AaeL_AAEL008167, AaeL_AAEL009691, AaeL_AAEL012576, AaeL_AAEL013613]
Arginine biosynthesis	0.0006491848	28.57	4	20.86	39.67	39.46	[AaeL_AAEL002399, AaeL_AAEL009872, AaeL_AAEL010464, AaeL_AAEL012579]
Alanine, aspartate and glutamate metabolism	0.0027175313	13.51	5	26.02	32.98	41.01	[AaeL_AAEL002399, AaeL_AAEL005422, AaeL_AAEL009872, AaeL_AAEL010464, AaeL_AAEL012579]
Cysteine and methionine metabolism	0.0468656942	10.34	3	0.00	40.13	59.87	[AaeL_AAEL002399, AaeL_AAEL012179, AaeL_AAEL012579]
Arginine and proline metabolism	0.0027175313	13.51	5	31.14	29.60	39.26	[AaeL_AAEL002399, AaeL_AAEL005422, AaeL_AAEL006834, AaeL_AAEL012579]
Tyrosine metabolism	0.0000740661	29.41	5	0.00	28.68	71.32	[AaeL_AAEL002399, AaeL_AAEL010442, AaeL_AAEL012579, AaeL_AAEL013637, AaeL_AAEL014600]
Phenylalanine metabolism	0.0001005801	44.44	4	0.00	33.45	66.55	[AaeL_AAEL002399, AaeL_AAEL010442, AaeL_AAEL012579, AaeL_AAEL014600]
Fatty acid degradation	0.0004754591	15.00	6	34.52	32.82	32.65	[AaeL_AAEL002296, AaeL_AAEL004739, AaeL_AAEL006634, AaeL_AAEL014452, AaeL_AAEL015524]
Valine, leucine and isoleucine degradation	0.0000513466	17.07	7	28.85	38.40	32.74	[AaeL_AAEL002296, AaeL_AAEL006634, AaeL_AAEL006928, AaeL_AAEL011137, AaeL_AAEL014452, AaeL_AAEL015524]
Lysine degradation	0.0583407879	8.11	3	34.52	32.82	32.65	[AaeL_AAEL002764, AaeL_AAEL006634, AaeL_AAEL015524]
Tryptophan metabolism	0.0384422913	11.54	3	34.52	32.82	32.65	[AaeL_AAEL006634, AaeL_AAEL015524, CAT1B]
Propanoate metabolism	0.0002085268	23.81	5	34.52	32.82	32.65	[AaeL_AAEL006634, AaeL_AAEL006928, AaeL_AAEL011746, AaeL_AAEL014452, AaeL_AAEL015524]
Butanoate metabolism	0.0017443846	22.22	4	23.15	44.02	32.84	[AaeL_AAEL006634, AaeL_AAEL011137, AaeL_AAEL015524]

**Table 5:** Proteins involved in the tyrosine metabolism pathway and their accession ID as shown in UniprotKB_AC.

ID	Protein	UniprotKB_AC
AaeL_AAEL012579	Aspartate aminotransferase (Position 29–397)	Q16LN3
AaeL_AAEL010442	AAEL010442-PA	Q16SY0
AaeL_AAEL014600	4-hydroxyphenylpyruvate dioxgenase	Q16FX9
AaeL_AAEL002399	Aspartate aminotransferase (Position 57–424)	Q5K6H3
AaeL_AAEL013637	Homogentisate 1,2-dioxygenase	Q16IJ1

## 4. Discussion

### 4.1. Effects of *B. pahangi* and *D. immitis* challenge on protein expression in *Ae. togoi*

In the present study, the whole proteome of *Ae. togoi* after filarial challenge (*D. immitis* and *B. pahangi*) was compared and identified for the first time in Malaysia and, by extension, Southeast Asia. A comparative proteomic analysis demonstrated significant differences in the expression of proteins/enzymes between filarial-challenged *Ae. togoi* and the control group. A total of 290 proteins were identified via LC/MS-MS and 192 proteins were expressed simultaneously in the three groups (DIM, BPH and control). Intriguingly, several immune proteins were discovered to be upregulated after infection with filarial parasites in the present study, which was also observed in another previous study where many of the immune proteins in *Ae. aegypti* that were found to be upregulated following infection with *D. immitis* encode secreted proteins. This indicates that the mosquito’s immune system mounts an active response to filarial infections by producing specific proteins, potentially aiding in controlling or limiting parasite development [16].

One of the recognised immunological mechanisms implicated in mosquitoes is larval melanisation, which restricts the growth of filarial parasites in mosquitoes was found in current study. The current data revealed that prophenoloxidase (PPO) was significantly increased in DIM. Although PPO was not substantially elevated in BPH, an increase was also detected in this group compared to the control. This humoral protein, when activated, could cause melanisation surrounding invading pathogens and concurrently stimulate cellular and humoral protection [17]. Given its participation in cellular and humoral defence, insect PPO is a vital innate immunity protein. It belongs to a group of copper-containing type-3 proteins found in almost all animals and eukaryotes species [18].

Over a century of research conducted on insect PPO has made the PPO activation cascade clearer. The insect PPO activation pathway involves several essential proteins, including pattern-recognition receptors (PGRP, GRP, and C-type lectins), serine proteases, and serine protease inhibitors (serpins) [19–21]. In insects, PPO activation is also essential for melanin production, which plays a role in preventing infection, aids in haemolymph clotting, and wound healing [22–25]. A specific phenoloxidase, PPO V, observed in *Armigeres subalbatus*, is associated with melanisation, blood feeding, and cuticle production in response to filarial parasites. Notably, this PPO showed considerable elevation following exposure to *D. immitis* microfilariae. In addition, silencing of PPO V in this investigation also led to a significant reduction in the degree of microfilariae melanisation, as well as in the rates of egg chronic melanisation and egg hatching in mosquitoes [26].

In addition, Shiao et al. reported the first direct evidence that PPO is necessary for successful melanisation of *D. immitis* by transducing *Ar. sulbabatus* mosquitoes with a double subgenomic Sindbis (dsSin) recombinant virus targeting the highly conserved copper-binding region of the phenoloxidase gene. This resulted in decreased of phenoloxidase activity in the transduced *Ar. subalbatus*. When these transduced mosquitoes were challenged with *D. immitis* microfilariae, melanisation of the microfilariae was almost completely inhibited [27]. All these investigations support the current findings, which demonstrated an increased in PPO expression after a filarial challenge. Although there was an increased of PPO expression post-filarial challenge and the first stage of melanised larvae is typically observed at 14 days post-infection [28], no melanised larvae were seen in the current study during the microscopic examination. This suggests that a process similar to immunological tolerance may be occuring both DIM and BPH in the current study.

Immune suppression in mosquitoes results from interference with the ability of circulating haemocytes to detect parasites (i.e., filarial and malaria parasites) as foreign or to be “activated” in response to the parasites [29]. Parasitoids may also actively disrupt some elements of the metabolic production of melanin, such as by inhibiting phenoloxidase activity [30], mobilisation of substrate precursors [31] and impairing normal haemocyte function [32]. Christensen and LaFond [33] first provided evidence that evidence of *B. pahangi-* infected *A. aegypti* has a reduced capacity for melanisation when challenged with intrathoracic inoculation of microfilariae. In addition, a recent study on the immunomodulatory effects of *B. malayi* microfilariae derived extracellular vesicles (EVs) on *Ae. aegypti* demonstrated that parasite-derived secreted EVs from *B. malayi* microfilariae were capable of interfering with critical immune responses in the vector host (*Ae. aegypti*), particularly immune responses such as melanisation [34]. Although filarial parasites can inhibit melanisation, it will not completely remove the melanisation response in mosquitoes [35]. This was also observed in the current study where there was an upregulation of PPO in DIM and BPH.

Consecutively, the expression fold change of actin in *Ae. togoi* was reported 2.55 and 2.34 times higher for DIM and BPH, respectively. These data revealed that the expression of actin is significantly elevated in both DIM and BPH which coincided with an earlier study that documented the upregulation of actin protein during infection and growth of the filarial parasites inside their vectors [36]. Actin is a highly versatile, ubiquitous and conserved protein which serves in a range of intracellular activities. Furthermore, actin acts in an antagonistic mechanism by restricting parasite infection in the gut tissue of mosquito. Likewise, a study also demonstrated an upregulation of the actin-1 gene in wild caught *An. gambiae* and *Cx. quinquefasciatus* infected with *W. bancrofti* [37].

Besides being upregulated after a filarial challenge in mosquitoes, actin was also found upregulated in *Drosophila melanogaster* larvae challenged by *Escherichia coli.* Functional genomic analysis proved participation of actin proteins in phagocytosis [23]. Remarkably, *An. gambiae* demonstrated the role of insect cytoplasmic actin as an extracellular pathogen recognition component that promotes antibacterial defence. Briefly, *An. gambiae* actin interacted with the extracellular MD2-like immune factor AgMDL1, and bound to the surface of bacteria (i.e., *E. coli*, *Enterobacter* bacterimum *(Esp_Z)*, *S. aureus* and *Bacillus pumilus*) which triggered phagocytosis, leading to bacterial death. The study also exposed *An. gambiae* to *Plasmodium falciparum*, where actin was identified as an antagonist of *P. falciparum* infection in the mosquito midgut. *Plasmodium falciparum* infection intensity increased by 2.7 log2 fold change after silencing the actin protein [38].

Another interesting protein identified in the current investigation was arginine kinase, which was upregulated—though not significantly—in both DIM and BPH groups. This protein has been shown to be upregulated in the brain of *Anopheles albimanus* during *Plasmodium berghei* infection [39]. Arginine kinase is a phosphotransferase which affects the nerve cell function and is involved in energy production. It can also mediate immune mechanism via alteration in the concentration of nitric oxide (NO) and the activity of iNOS, which has also been reported in scallop (*Chlamys farreri*) [40]. In addition, NO is a notable immune stress mediator, associated in systemic immune response signalling pathway which is released during immunological responses and is also a neuromodulator coordinating numerous neuronal activities process in insects (i.e., *Schistocerca gregaria* and *Manduca sexta*) [41–43]. However, up to the present known published studies, limited information has been conducted on arginine kinase in mosquito infected with filarial parasites. From all the investigations done on different species of insect afflicted with different species of parasites, arginine kinases may be theorised to serve a degree of role in modifying the immunological reaction via variations in the concentration of NO in the present investigation. Hence, additional research may be required to uncover the significance of the arginine kinase in mosquitoes towards the infection of filarial parasites.

On the other hand, transferrin, another protein implicated in mosquito immune responses, was found to be not significantly downregulated in either of the filarial-inoculated groups. A previous study observed that transferrin levels increased 24 hours post-infection in *Ae. aegypti* and *Cx. quinquefasciatus* infected with *W. bancrofti*, compared to uninfected controls, though no significant difference in transcript levels was found at 3 or 7 days post-infection [44]. This contrasts with the current study, where transferrin showed no significant downregulation. In vertebrates, transferrin functions as an iron-binding protein that plays a key function in iron transport. This molecule is released in the haemolymph of insects involved in iron transport, and functions as potent immune proteins, antioxidants, vitellogenin proteins and it is also a part of the humoral immune system, potentially acting as an antibiotic agent [45–48]. Oxidative stress also contributes to the increased of transferrin in mosquitoes. Mosquitoes face oxidative stress due to the release of iron from haemoglobin after the iron-rich blood meal [49].

Previously, an 84-kDA protein was upregulated after *B. pahangi* larvae was melanotically encapsulated in *Ae. aegypti* [50] but it was later determined to be transferrin [45]. In addition, a prominent protein band at approximately 66 kDa was observed in the SDS-PAGE analysis of *Cx. quinquefasciatus* specimens collected from filariasis-endemic areas in a previous study [51]. The hazardous reactive intermediates released during melanin biosynthesis of filarial worm encapsulation may also induce oxidative stress in insects [52]. Transferrin appears to be expressed under settings of oxidative stress, in addition to being upregulated by pathogen infection. The results of the present study contradict those of previous research, which may be attributed to specific factors that triggered downregulation of transferrin in response to filarial challenge. Given the overlap in molecules involved in immune responses, it would be valuable to determine whether the presence of the parasite triggers stress signals or whether the mechanisms to control/eliminate the pathogen stimulate transferrin production in such scenarios [52,53]. In addition, it is also important to explore whether transferrin can influence pathogen infection in mosquitoes against various pathogens (i.e., bacteria, filarial worms or other diseases) [44].

Two other noteworthy proteins that were identified in the current investigation were Vitellogenin B and Vitellogenin C. Vitellogenin B was significantly elevated in DIM but significantly downregulated in BPH, whereas Vitellogenin C showed significant upregulation in both groups. Vitellogenin is widely recognised as a female-specific protein but it also has been observed in male insects (i.e., *Leucophaea maderae* and *Nicrophorus vespilloides*) [54–55] and juvenile insects (*Apis mellifera*) [56], indicating that its role extends beyond serving as an energy source for developing embryos [57]. A few studies also observed that vitellogenin contains immunologic characteristics by conjugating to the pathogens and neutralising or eliminating the pathogens (bacteria, viruses and fungus) either directly or indirectly as antibody-mediated macrophage phagocytosis occurs in various fish species [58–60].

A previous study also demonstrated that vitellogenin in *Danio rerio* (zebrafish) serum could bind to both *E. coli* and *S. aureus*, inhibiting bacterial growth. This study conclusively showed that vitellogenin acts as an acute-phase protein with bacterial-binding and inhibitory actions in zebrafish [61]. In addition, vitellogenin was also responsible for reducing the parasite-killing efficacy in *An. gambiae* of the antiparasitic factor TEP1 against *P. falciparum* [62]. Although no research has been conducted on the immunologic properties of vitellogenin in mosquitoes against filarial parasites, all prior studies support the current study’s hypothesis that vitellogenin might be involved in the mosquitoes’ immune responses against filarial parasites.

Among the proteins that were found in control but absent in both DIM and BPH, calcium-transporting ATPase which is associated with viral replication was also found in this study. Calcium-transporting ATPase proteins of the sarco (endo) plasmic reticulum (SERCA), plasma membrane (PMCA), and secretory pathway (SPCA) are essential for muscle function, calcium cell signalling, calcium transport into secretory vesicles, mitochondrial function, and the induction of apoptosis [63–65]. Both in vitro and in vivo studies have shown that calcium-transporting ATPases in Ae. aegypti are involved in dengue virus replication. Inhibition of calcium-transporting ATPase of *Ae. aegypti* may result in significant reduction in the infection rate of dengue virus. There is a substantial positive correlation between the amount of calcium-transporting ATPase in mosquitoes and the virulence of dengue virus [66]. Although this protein was not detected in the current study, previous findings suggest that targeting specific mosquito proteins like calcium-transporting ATPase may offer a potential strategy for dengue virus control.

### 4.2. Effects of *D. immitis* and *B. pahangi* challenge on metabolic changes in *Ae. togoi*

Most enzymes/proteins involved in glycolysis and gluconeogenesis were increased in *Ae. togoi* infected with filarial worms. This may be attributed to the increased expression of glycolytic genes caused by the higher energy consumption of active immune systems. Immunity is one of the most fundamental evolutionary characteristics that all creatures, including insects, possess. In most circumstances, organisms possess a range of pre-existing, innately present immune systems that are ready to function immediately. These systems stay dormant until activated by a pathogen, spending just little more energy than is necessary for their fundamental operation (maintenance costs) [66].

In insects, energy is derived from carbohydrate sources such as glycogen, which is enzymatically converted into glucose and subsequently used to produce high levels of ATP through glycolysis and the TCA cycle [67,68]. When energy is required, glycogen can be converted to glucose and enter the glycolysis-tricarboxylic acid (glycolysis-TCA) cycle. In glycolysis, glucose serves as the initial substrate, and a sequence of enzymes, including hexokinase (HK), phosphofructokinase (PFK), and pyruvate kinase, convert glucose into pyruvate (PK). Gluconeogenesis is the reverse process, and fructose-1,6-bisphosphatase (FBP) is involved in this reaction. Pyruvate can be transformed into acetylcoenzyme A (acetyl-CoA), recycled in the TCA pathway, and then oxidised to provide energy as CO_2_ and H_2_O. The enzymes diphosphopyridine nucleotide-dependent malic enzyme and triphosphopyridine nucleotide-dependent malic enzyme may regenerate pyruvate from malate, the intermediate in TCA [69].

In *Drosophila* larvae, for instance, the growth and maintenance of haematopoietic cells, which are essential for cellular immunity, require around 10% of the organism’s total glucose [70]. Depending on the severity of the reaction, the immune system’s energy consumption skyrocket when it is triggered. Immune response induction is associated with deployment costs [67]. For instance, initiating immune cell migration and phagocytosis demands more energy [71]. In contrast, the detection of a pathogen initiates a cascade of further immune responses requiring the quick synthesis of multiple new molecules with signalling and antimicrobial properties, the fulfilment of different cellular tasks, and the proliferation of extra immune cells (for example, lamellocytes for encapsulation).

Consequently, immune cell energy needs may increase from 10 percent to almost one-third of total glucose consumption, as seen in the case of *Drosophila* larvae developing an immune response to a parasitoid infection [70]. Immune cells undergo a metabolic change during activation of cellular immunity, becoming dependent on a significant supply of glucose and glutamine [72]. This metabolic reprogramming is unquestionably necessary for anti-pathogen responses, as deprivation or inhibition of the signal transduction for metabolic reprogramming has been shown to diminish the immune response to infections [70,73,74].

Although it is abundantly clear that the activation of various insect immune cells leads to a substantial increase in glucose consumption and glycolysis, it is essential to study the metabolic changes of mosquitoes in order to understand the relationship between the research conducted on mosquitoes following a filarial challenge and studies conducted in mammals (e.g.,., humans and dogs) infected with filarial parasites.

### 4.3. Role of tyrosine in melanisation of filarial parasites in mosquitoes

In the present study, five proteins/enzymes (aspartate aminotransferase position 29–397 and position 56–424, AAEL010442-PA, 4-hydroxyphenylpyruvate dioxygenase and homogentisate 1,2-dioxygenase) which are involved in the synthesis of tyrosine were uniquely expressed in DIM and whereby only 2 of the proteins/enzymes (aspartate aminotransferase protein position 29–397 and aspartate aminotransferase protein position 57–424) were differentially expressed in BPH compared to control. It has been known for a long time that insects depend substantially on tyrosine for cuticle thickening and melanisation of pathogens which are crucial components of the insect immune system. As a result, insects carry more tyrosine metabolic enzymes than humans do [75]. Tyrosine storage is the accumulation of tyrosine-rich proteins in the haemolymph of many insects [76], and the formation of tyrosine-loaded vacuoles in the fat body of other insects [77]. Additionally, tyrosine levels have been found to be significantly increased in *Ar. subalbatus* infected with *B. malayi* [78].

Another study, conducted by Fuchs et al. [79], silenced the gene of phenylalanine hydroxylase (PAH), which is involved in the conversion of phenylalanine to tyrosine. This intervention resulted in increased levels of phenylalanine, phenylpyruvate, and phenylactate. Interestingly, the amount of tyrosine available for melanin synthesis was reduced and resulted in significant impairment of melanotic encapsulation response against *Plasmodium berghei*. All these findings were relevant and offered strong evidence for the current investigation, in which five proteins involved in tyrosine metabolism were expressed in BPH (aspartate aminotransferase protein position 29–397 and aspartate aminotransferase protein position 57–424) and DIM (aspartate aminotransferase position 29–397 and position 56–424, AAEL010442-PA, 4-hydroxyphenylpyruvate dioxygenase and homogentisate 1,2-dioxygenase).

## 5. Conclusion

In conclusion, the proteins known to be involved in immune regulation and metabolic activities of mosquitoes were identified in the current study. The predominance of glycolysis-TCA cycle and gluconeogenesis-related enzymes/proteins were elevated in both DIM and BPH. Additionally, the expression of five proteins involved in tyrosine production was detected in the current study. Overall, the findings of this study underscore the intricate interplay between immune regulation, metabolism, and parasitic infection in mosquitoes, shedding light on potential targets for future intervention strategies against the neglected tropical filarial diseases.

## Supporting information

S1 Table Upregulated proteins of BPH and DIM compared to control. (DOCX)

S2 Table Downregulated proteins of BPH and DIM compared to control. (DOCX)

S3 Table Inconsistent upregulated and downregulated proteins of BPH and DIM compared to control. (DOCX)

S4 Table Proteins exclusively identified BPH and DIM. (DOCX)

S5 Table Protein exclusively identified in DIM. (DOCX)

S6 Table Protein exclusively identified in BPH. (DOCX)

S7 Table Proteins exclusively identified in control. (DOCX)

S8 Table Downregulated proteins of DIM compared to control. (DOCX)

S9 Table Upregulated and downregulated proteins of BPH compared to control. (DOCX)

S10 File Raw Data of LC/MS-MS. (XLSX)
