# Supplementary material for: Proteomic analysis unveils host-parasite interactions in Aedes togoi infected with Dirofilaria immitis and Brugia pahangi
Source: PLoS One. 2025 Jul 9;20(7):e0326693. doi: 10.1371/journal.pone.0326693 (PMC12240324; doi:10.1371/journal.pone.0326693)
Supplement: S1 Table — (DOCX) [file pone.0326693.s001.docx]

**Table S1. Upregulated proteins of BPH and DIM compared to control**

| **No** | **Protein** | **BPH** | | **DIM** | | **Protein IDs** |
| --- | --- | --- | --- | --- | --- | --- |
|  |  | **Fold change** | **log2Fold change** | **Fold change** | **log2Fold change** |  |
|  | Phosphofructo-2-kinase | 17.50 | 4.13 | 30.94 | 4.95 | A0A6I8TF90;A0A6I8TNE7;A0A6I8TNB3;Q16K25;Q16K24 |
|  | Fructose-bisphosphatase | 12.61 | 3.66 | 32.05 | 5.00 | Q17M22;Q17M23 |
|  | 40S ribosomal protein SA | 10.41 | 3.38 | 14.20 | 3.83 | A0A6I8TLV1;Q16ZR8 |
|  | Glyceraldehyde-3-phosphate dehydrogenase | 8.51 | 3.09 | 14.74 | 3.88 | J9HYM2;A0A292AH36 |
|  | Elongation factor 1-alpha | 6.81 | 2.77 | 8.23 | 3.04 | Q1HR88;Q5MM87;J9HYQ9 |
|  | Phosphate carrier protein, mitochondrial | 6.54 | 2.71 | 2.24 | 1.16 | Q16QS3;Q16QS2;Q1HQX9 |
|  | 40S ribosomal protein S2 | 6.50 | 2.70 | 5.01 | 2.32 | Q1HRV1;Q5QC97 |
|  | Isocitrate dehydrogenase NAD subunit, mitochondrial | 5.59 | 2.48 | 4.31 | 2.11 | Q17P80;A0A1S4EVW3;Q17P79;A0A1S4EVV7 |
|  | 60S ribosomal protein L4 | 5.36 | 2.42 | 7.35 | 2.88 | Q1HQJ0 |
|  | Uncharacterized protein | 5.34 | 2.42 | 7.71 | 2.95 | Q16IL6;A0A6I8TFM3;A0A6I8TQI9 |
|  | Ubiquitin-activating enzyme E1 | 5.20 | 2.38 | 10.81 | 3.44 | Q17N85;Q17N86 |
|  | Pyruvate dehydrogenase E1 component subunit beta | 4.83 | 2.27 | 5.86 | 2.55 | Q17D51;A0A1S4F7M2 |
|  | Lamin | 4.77 | 2.26 | 4.71 | 2.23 | Q17FG6;A0A6I8T7Y3 |
|  | AAEL010205-PA | 4.60 | 2.20 | 4.77 | 2.25 | Q16TK2;Q16TK1 |
|  | 26S proteasome non-ATPase regulatory subunit 2 | 4.50 | 2.17 | 6.28 | 2.65 | Q176M6 |
|  | MICOS complex subunit MIC60 | 4.26 | 2.09 | 5.79 | 2.53 | Q16U68;Q16U69;A0A1S4FNY8;A0A6I8TCN5 |
|  | Sodium/potassium-transporting ATPase subunit alpha | 4.12 | 2.04 | 6.31 | 2.66 | Q16N76;Q16N75;Q16N74;A0A6I8TLQ1;A0A6I8TLL6 |
|  | 1,4-alpha-glucan branching enzyme | 3.82 | 1.93 | 10.37 | 3.37 | Q16SE5;Q16PC7 |
|  | ATP synthase subunit gamma | 3.82 | 1.93 | 8.36 | 3.06 | Q16XK3 |
|  | 26S proteasome non-ATPase regulatory subunit 6 | 3.80 | 1.92 | 5.94 | 2.57 | Q16U24;A0A1S4FP45 |
|  | Actin | 5.07 | 2.34 | 5.84 | 2.55 | Q1HRN5;Q6ELZ6;Q17C86 |
|  | AAEL017262-PA | 3.72 | 1.90 | 3.37 | 1.75 | J9HFG0 |
|  | 40S ribosomal protein S8 | 3.66 | 1.87 | 5.11 | 2.35 | Q1HRQ9;Q0IER3 |
|  | AAEL010403-PA Past-1 | 3.46 | 1.79 | 9.41 | 3.23 | Q16T24;A0A6I8TCY9 |
|  | Dynamin-type G domain-containing protein | 3.41 | 1.77 | 7.64 | 2.93 | Q17GK8;A0A6I8U8P6;A0A0N8ES72;A0A6I8U646 |
|  | Lethal(2)essential for life protein | 3.27 | 1.71 | 3.30 | 1.72 | Q16S81;Q16JG7 |
|  | AAEL013612-PA | 3.20 | 1.68 | 1.85 | 0.89 | Q16IL6;A0A6I8TFM3;A0A6I8TQI9 |
|  | Glucose-6-phosphate 1-dehydrogenase | 3.14 | 1.65 | 10.97 | 3.46 | A0A6I8THL2;Q0IEL8 |
|  | Eukaryotic translation initiation factor 3 subunit M | 3.08 | 1.62 | 5.65 | 2.50 | Q17D30 |
|  | Heat shock protein 83 | 3.07 | 1.62 | 5.16 | 2.37 | Q16PB5;Q16FA5;A0A1S4FUH2;Q16FA6 |

**Table S1. Continued…**

| **No** | **Protein** | **BPH** | | **DIM**  **Protein** | | **Protein IDs** |
| --- | --- | --- | --- | --- | --- | --- |
|  |  | **Fold change** | **log2Fold change** | **Fold change** | **log2Fold change** |  |
|  | Transmembrane trafficking protein GOLD domain-containing protein | 3.00 | 1.58 | 6.92 | 2.79 | Q176Q6;Q1HQX4;A0A6I8TZL9 |
|  | RNA helicase | 3.03 | 1.60 | 9.44 | 3.24 | Q16YL3 |
|  | 60S ribosomal protein L8 | 2.98 | 1.57 | 4.03 | 2.01 | Q1HR32 |
|  | FACT complex subunit | 2.95 | 1.56 | 4.11 | 2.04 | Q16EH0;Q16NS9;A0A6I8U9W3 |
|  | 60S ribosomal protein L5 | 2.92 | 1.55 | 10.59 | 3.40 | Q1HQU2 |
|  | Eukaryotic translation initiation factor 3 subunit | 2.92 | 1.55 | 4.13 | 2.05 | Q0IEY3;A0A6I8TEN4 |
|  | AAEL004565-PA | 2.82 | 1.50 | 1.30 | 0.37 | Q17CH1;A0A6I8U3S3 |
|  | Fructose-bisphosphate aldolase | 2.79 | 1.48 | 3.64 | 1.86 | Q178U9;Q178U8 |
|  | Chaperonin-60kD | 2.75 | 1.46 | 4.23 | 2.08 | Q16PM9 |
|  | Vitellogenin-C | 6.48 | 2.70 | 4.33 | 2.12 | Q177I4;Q6UBM1;Q177I5;A0A1S4FCM3 |
|  | Eukaryotic translation initiation factor 3 subunit B | 2.69 | 1.42 | 3.88 | 1.96 | Q0IEY3;A0A6I8TEN4 |
|  | Histone H2A | 2.58 | 1.37 | 4.64 | 2.21 | Q16LW9 |
|  | Glycerol-3-phosphate dehydrogenase NAD(+) | 2.56 | 1.36 | 6.14 | 2.62 | Q17KS3;A0A6I8T5F9;Q17KS5;Q17KS4;A0A1S4EZ75 |
|  | Tubulin alpha chain | 2.52 | 1.33 | 3.71 | 1.89 | Q1HR53;Q16JS3 |
|  | Activated protein kinase C receptor Guanine nucleotide-binding protein subunit beta-like protein | 2.51 | 1.33 | 6.75 | 2.76 | Q1HRQ2 |
|  | Eukaryotic translation initiation factor 3 subunit A | 2.48 | 1.31 | 5.39 | 2.43 | Q173M7 |
|  | Heparan n-sulfatase | 2.47 | 1.30 | 2.59 | 1.38 | Q16T02;A0A0N8ES60;A0A1S4FQA7 |
|  | NADH dehydrogenase [ubiquinone] 1 beta subcomplex subunit 9 | 2.44 | 1.28 | 1.01 | 0.01 | Q16TI5 |
|  | 40S ribosomal protein S3a | 2.37 | 1.25 | 3.95 | 1.98 | Q0IFA5;Q1HRR3 |
|  | ATP-dependent 6-phosphofructokinase | 2.27 | 1.18 | 6.68 | 2.74 | Q174J0;A0A6I8TDP2;A0A6I8TDB1 |
|  | Alpha-1,4 glucan phosphorylase | 2.26 | 1.18 | 4.47 | 2.16 | Q17NG8 |
|  | 60S ribosomal protein L18 | 2.18 | 1.12 | 4.56 | 2.19 | Q1HR62 |
|  | Heme peroxidase | 2.17 | 1.12 | 2.98 | 1.58 | Q17CY7;A0A1S4F7G0 |
|  | Acetyl-coa acetyltransferase, mitochondrial (acetoacetyl-coa thiolase) | 2.15 | 1.11 | 4.11 | 2.04 | Q175J7;A0A1S4FE42 |
|  | Prophenoloxidase | 2.14 | 1.10 | 3.04 | 1.60 | Q16IZ1 |
|  | Ras-related protein Rab-2A | 2.06 | 1.04 | 2.64 | 1.40 | Q17BP4 |
|  | 14-3-3 protein zeta | 2.05 | 1.03 | 3.75 | 1.91 | Q1HR36;A0A6I8TEM3 |
|  | Signal sequence receptor subunit delta | 2.02 | 1.01 | 2.36 | 1.24 | Q16JI4 |
|  | AAEL004249-PB | 1.99 | 1.00 | 1.55 | 0.63 | Q17DD5;Q17DD4 |
|  | Cytochrome c oxidase polypeptide Va | 1.92 | 0.94 | 1.34 | 0.43 | Q1HRJ0;Q16Q43 |
|  | Alpha-mannosidase (Fragment) | 1.89 | 0.92 | 2.32 | 1.22 | Q178V9;A0A6I8TCS3;A0A6I8TBJ2;Q178W0;A0A1S4FBS2 |

**Table S1. Continued…**

| **No** | **Protein** | **BPH** | **DIM**  **Protein** | **DIM**  **Protein** | **Protein** | **Protein IDs** |
| --- | --- | --- | --- | --- | --- | --- |
|  |  | **Fold change**  **log2Fold change** | **Fold change**  **log2Fold change** | **Fold change** | **log2Fold change** |  |
|  | Transketolase | 1.73 | 0.80 | 4.60 | 2.20 | Q17CT0 |
|  | Proteasome subunit beta | 1.79 | 0.84 | 3.79 | 1.92 | Q16JS7;A0A1S4FYT3 |
|  | 60S ribosomal protein L17 | 1.76 | 0.82 | 1.25 | 0.32 | Q1HR65 |
|  | Serine hydroxymethyltransferase | 1.67 | 0.74 | 3.28 | 1.71 | Q17HZ9;A0A1S4F1X0;Q17I00;A0A1S4F1Y3 |
|  | 6-phosphogluconate dehydrogenase, decarboxylating | 1.52 | 0.61 | 2.91 | 1.54 | Q178E4;A0A6I8TC18 |
|  | RNA helicase | 1.51 | 0.60 | 3.56 | 1.83 | Q16JE7;Q1HR82 |
|  | AAEL001061-PB | 1.50 | 0.59 | 2.92 | 1.54 | J9HHL7;J9HXZ8;A0A1S4EXN8;A0A1S4EXN6;Q17MA9 |
|  | Catalase | 1.39 | 0.48 | 1.90 | 0.93 | J9HGP0;Q1HRH7 |
|  | Isocitrate dehydrogenase [NADP] | 1.38 | 0.47 | 2.45 | 1.29 | Q16LK8;Q1HQW5;A0A6I8U913;A0A0P6IWM1 |
|  | Enoyl-CoA hydratase | 1.37 | 0.45 | 3.58 | 1.84 | Q1DGR4;Q16TS7;A0A6I8TIN5 |
|  | Pyruvate carboxylase | 1.37 | 0.45 | 4.56 | 2.19 | Q16V52;A0A6I8TI71;Q16921 |
|  | Glycogen starch synthase | 1.37 | 0.45 | 3.52 | 1.82 | Q17DG0 |
|  | Pupal cuticle protein, putative | 1.35 | 0.43 | 1.71 | 0.78 | Q16IX7;A0A1S4FZ80 |
|  | Trifunctional enzyme beta subunit (tp-beta) | 1.34 | 0.42 | 2.48 | 1.31 | Q17IM1 |
|  | Clathrin heavy chain | 1.34 | 0.42 | 2.98 | 1.58 | Q16IM0;A0A6I8U1U7;A0A6I8U540 |
|  | Dihydrolipoyllysine-residue succinyltransferase component of 2-oxoglutarate dehydrogenase complex, mitochondrial | 1.33 | 0.41 | 1.51 | 0.59 | Q17H89;A0A6I8T705 |
|  | AAEL002372-PA | 1.32 | 0.40 | 2.49 | 1.32 | Q17IE1;Q9GSB1 |
|  | AAEL007698-PA | 1.31 | 0.39 | 2.52 | 1.34 | Q171B3 |
|  | ATP synthase subunit b | 1.29 | 0.36 | 1.95 | 0.97 | Q179J9 |
|  | Proteasome subunit alpha type | 1.28 | 0.36 | 1.79 | 0.84 | Q16TY4 |
|  | AAEL009955-PA | 1.27 | 0.35 | 3.38 | 1.76 | Q16UB8;A0A6I8TK86 |
|  | Glutaminyl-tRNA synthetase | 1.27 | 0.34 | 2.04 | 1.03 | Q16Q55 |
|  | Dynein heavy chain | 1.27 | 0.34 | 3.75 | 1.91 | Q173I7;A0A6I8TDM5;A0A6I8TDN4;A0A6I8TE32 |
|  | 40S ribosomal protein S13 | 1.25 | 0.32 | 1.69 | 0.76 | Q5QC94 |
|  | 2-Cys thioredoxin peroxidase | 1.24 | 0.30 | 1.18 | 0.24 | Q17DN4;Q8WSF6 |
|  | GTP-binding protein alpha subunit, gna | 1.22 | 0.29 | 1.39 | 0.48 | Q16SS3;A0A6I8TJF8 |
|  | Catalase | 1.17 | 0.23 | 1.25 | 0.32 | Q16J86 |
|  | Glutamate dehydrogenase (NAD(P)(+) | 1.15 | 0.20 | 1.39 | 0.47 | Q5QC94 |
|  | Glycerol-3-phosphate dehydrogenase | 1.13 | 0.18 | 1.32 | 0.41 | Q17E81;Q17E82 |
|  | ATP synthase subunit alpha | 1.13 | 0.18 | 1.23 | 0.29 | Q1HRQ7 |
|  | Alanine--tRNA ligase | 1.10 | 0.14 | 1.23 | 0.30 | Q17LY0 |

**Table S1. Continued…**

| **No** | **Protein** | **BPH** | | **DIM** | | **Protein IDs** |
| --- | --- | --- | --- | --- | --- | --- |
|  |  | **Fold change** | **log2Fold change** | **Fold change** | **log2Fold change** |  |
|  | Polyadenylate-binding protein | 1.05 | 0.07 | 1.84 | 0.88 | Q1HR66 |
|  | Mitochondrial medium-chain acyl-CoA dehydrogenase | 1.10 | 0.14 | 1.83 | 0.87 | Q16GA1;Q1HQM4 |
|  | Proteasome subunit alpha type | 1.09 | 0.12 | 1.41 | 0.49 | Q16TY4 |
|  | AAEL017349-PA | 1.07 | 0.10 | 1.68 | 0.75 | Q1HR69 |
|  | AAEL007023-PA | 1.04 | 0.06 | 4.00 | 2.00 | Q173X5 |
|  | Mitochondrial porin Voltage-dependent anion channel | 1.03 | 0.05 | 1.02 | 0.03 | Q1HR57 |
|  | NADH-ubiquinone oxidoreductase 49 kDa subunit | 1.01 | 0.01 | 1.08 | 0.10 | Q16Q68 |
|  | Arginine kinase | 1.63 | 0.71 | 1.74 | 0.80 | Q1HR67;A0A2I7ML30;A0A6I8TIX2;A0A6I8TBR6 |
|  | Nucleoside diphosphate kinase | 1.11 | 0.15 | 1.09 | 0.13 | Q1HRN9;Q16MB4;A0A6I8U5H3 |
|  | Thioredoxin Peroxidase | 1.24 | 0.31 | 1.18 | 0.24 | Q17DN4;Q8WSF6 |
|  | Bacteria-responsive protein 1 AgBR1 | 2.75 | 1.46 | 1.38 | 0.47 | Q1HRE7;Q17JL7;A0A1S4F0I0 |
|  | Imaginal disc growth factor | 2.26 | 1.18 | 1.47 | 0.55 | A0A1S4F0F9;Q17JL4;Q5MM84 |

| Colour code |  |
| --- | --- |
|  | Significantly expressed (Log2Fold change= >3.0) |
|  | Significantly expressed (Log2Fold change= >2.0, <3.0) |
|  | Significantly expressed (Log2Fold change= >1.5, <2.0) |
|  | Not significantly expressed (Log2Fold change= >1.0, <1.5) |
|  | Not significantly expressed (Log2Fold change= >0.5, <1.0) |
|  | Not significantly expressed (Log2Fold change= <0.5) |
