# Supplementary material for: Proteomic analysis unveils host-parasite interactions in Aedes togoi infected with Dirofilaria immitis and Brugia pahangi
Source: PLoS One. 2025 Jul 9;20(7):e0326693. doi: 10.1371/journal.pone.0326693 (PMC12240324; doi:10.1371/journal.pone.0326693)
Supplement: S2 Table — (DOCX) [file pone.0326693.s002.docx]

**Table S2. Downregulated proteins of BPH and DIM compared to control**

| **No** | **Protein** | **BPH** | | **DIM** | | **Protein IDs** |
| --- | --- | --- | --- | --- | --- | --- |
|  |  | **Fold change** | **log2Fold change** | **Fold change** | **log2Fold change** |  |
|  | PKS_ER domain-containing protein | -253.17 | -7.98 | -43.70 | -5.45 | Q16S95;A0A6I8TU49 |
|  | Tropomyosin invertebrate | -28.37 | -4.83 | -16.36 | -4.03 | Q17H71;Q17H76;A0A6I8T6W0;A0A6I8T7F4;A0A6I8T7F9 |
|  | NADH dehydrogenase ubiquinone flavoprotein 1, mitochondrial | -17.38 | -4.12 | -1.03 | -0.04 | Q1HR11;Q171D1 |
|  | NADH-ubiquinone oxidoreductase 24 kda subunit | -12.86 | -3.68 | -2.65 | -1.41 | Q1HRL6;Q179U9;Q5UG10 |
|  | Spectrin beta chain | -11.66 | -3.54 | -3.16 | -1.66 | Q178J6 |
|  | Myosin heavy chain, nonmuscle or smooth muscle | -10.62 | -3.41 | -2.85 | -1.51 | Q179E8 |
|  | AAEL013559-PA | -10.32 | -3.37 | -4.23 | -2.08 | Q16IU1;A0A6I8TT30 |
|  | Dihydrolipoyl dehydrogenase | -7.51 | -2.91 | -5.36 | -2.42 | Q174D6;A0A1S4FF23 |
|  | AAEL011551-PA | -7.42 | -2.89 | -5.95 | -2.57 | Q16PR4;A0A1S4FTI5 |
|  | NADH-ubiquinone oxidoreductase 75 kDa subunit, mitochondrial | -5.75 | -2.53 | -3.67 | -1.87 | Q16LR5;A0A1S4FWT8 |
|  | Spectrin | -4.43 | -2.15 | -3.05 | -1.61 | Q16EQ1;A0A6I8TS46;A0A6I8TGL5;A0A6I8TQK0 |
|  | Paramyosin, long form | -4.23 | -2.08 | -4.42 | -2.14 | Q16RF4 |
|  | Vacuolar ATP synthase subunit E | -4.11 | -2.04 | -4.46 | -2.16 | Q1HQT6 |
|  | Fumarate hydratase | -4.01 | -2.00 | -1.62 | -0 .70 | Q16ZK9;A0A6I8TFZ7;A0A6I8TFH2;Q16ZL0 |
|  | Myosin heavy chain, nonmuscle or smooth muscle | -3.43 | -1.78 | -2.38 | -1.25 | Q179E8 |
|  | NADH-ubiquinone oxidoreductase 39 kda subunit | -3.37 | -1.75 | -1.72 | -0.78 | Q16YE9;A0A1S4FJU4 |
|  | Alanine aminotransferase | -3.37 | -1.75 | -1.29 | -0.37 | Q16UK8;A0A1S4FNL0;Q16UK6;Q16UK7;A0A6I8U8Z9 |
|  | Alpha-actinin | -3.09 | -1.63 | -1.99 | -0.99 | Q172T4;A0A6I8T9W6 |
|  | V-type proton ATPase catalytic subunit A | -3.05 | -1.61 | -1.21 | -0.27 | O16109 |
|  | Muscle-specific actin 2 | -3.03 | -1.60 | -5.63 | -2.49 | Q17KG3;Q45L89;Q6ELZ7 |
|  | ATP synthase subunit beta | -2.84 | -1.50 | -1.66 | -0.73 | Q17H12;Q17FL3;A0A1S4F4H1;Q1HR61 |
|  | Succinate dehydrogenase ubiquinone iron-sulfur subunit, mitochondrial | -2.66 | -1.41 | -1.86 | -0.90 | Q16TA7 |
|  | Acetyl-CoA deacylase | -2.63 | -1.40 | -1.25 | -0.32 | Q0IG02 |
|  | AAEL005324-PA | -2.61 | -1.38 | -1.63 | -0.70 | Q17AE9;A0A6I8TJB7 |
|  | AAEL005435-PA | -2.53 | -1.34 | -2.58 | -1.37 | Q17A09 |
|  | NADH-ubiquinone oxidoreductase 39 kda subunit | -2.50 | -1.32 | -1.14 | -0.19 | Q16VZ4 |
|  | Complex I-B14 | -2.26 | -1.17 | -1.35 | -0.43 | Q16KC4;Q17Q29 |
|  | Putative 56.5 kDa secreted protein (Fragment) | -2.18 | -1.13 | -2.19 | -1.13 | Q8T9V3;Q58HB4 |
|  | Histone H2A | -2.10 | -1.07 | -1.66 | -0.73 | Q17ER8;Q17ER3;Q17EF0;Q173V2;A0A6I8TYT7;Q171J5 |
|  | 26S proteasome non-ATPase regulatory subunit 1 | -2.04 | -1.03 | -3.04 | -1.60 | Q17GS7;A0A1S4F345 |

**Table S2. Continued…**

| **No** | **Protein** | **BPH** | | **DIM** | | **Protein IDs** |
| --- | --- | --- | --- | --- | --- | --- |
|  |  | **Fold change** | **log2Fold change** | **Fold change** | **log2Fold change** |  |
|  | Histone H4 | -2.00 | -1.00 | -1.28 | -0.35 | Q17EE8;Q16IE4;A0A6I8U2Q5;A0A6I8U427;A0A6I8U625 |
|  | Probable citrate synthase 1, mitochondrial | -1.99 | -0.99 | -1.42 | -0.50 | Q17GM7;Q16P20 |
|  | Troponin I | -1.80 | -0.85 | -5.57 | -2.48 | Q16RS1;Q16RS9;Q16RS5 |
|  | ATP synthase subunit d Vacuolar H+-ATPase V1 sector subunit D | -1.67 | -0.74 | -1.18 | -0.24 | Q1HQU5 |
|  | RNA helicase | -1.66 | -0.73 | -9.29 | -3.22 | Q17KA8;A0A1S4EZS3;A0A6I8T693;Q17II7;Q16T16 |
|  | AAEL011180-PA | -1.42 | -0.51 | -2.18 | -1.13 | Q16QU2 |
|  | Pyruvate dehydrogenase E1 component subunit alpha | -1.34 | -0.42 | -1.68 | -0.75 | Q16IL3 |
|  | Proteasome subunit alpha type | -1.31 | -0.39 | -1.05 | -0.07 | Q1HQN1 |
|  | Nidogen | -1.31 | -0.39 | -1.20 | -0.26 | Q172X3;A0A1S4FG03 |
|  | Putative mitochondrial atp synthase epsilon chain | -1.31 | -0.39 | -1.18 | -0.24 | Q17DS2;Q17DS3;A0A0P6IZV0 |
|  | Opioid-binding proteincell adhesion molecule, putative | -1.29 | -0.36 | -1.46 | -0.54 | Q17DI1;A0A6I8T6Y2;A0A6I8TA10;A0A6I8T959 |
|  | AAEL012400-PA | -1.24 | -0.31 | -1.60 | -0.68 | Q16M72;A0A6I8TIS5 |
|  | Prohibitin | -1.19 | -0.26 | -1.04 | -0.05 | Q1HR13 |
|  | Annexin | -1.10 | -0.13 | -1.50 | -0.58 | Q17A52;A0A6I8T820;Q17A53 |
|  | Guanine nucleotide-binding protein beta 3 (g protein beta3) | -1.09 | -0.13 | -4.76 | -2.25 | Q16HK0;Q16HK1;Q17MH7 |
|  | Putative adult cuticle protein 1 | -1.05 | -0.07 | -1.17 | -0.23 | A0A6I8TTY0;A0A0P6IY13 |
|  | Glutathione transferase | -1.04 | -0.05 | -16.41 | -4.04 | Q16P79;A0A6I8TE01;A0A6I8TLA6 |
|  | Acyl-coa dehydrogenase | -2.19 | -1.13 | -1.27 | -0.35 | Q17DJ8;A0A1S4F6S3;Q17DJ9 |
|  | CCT-beta | -1.71 | -0.78 | -1.19 | -0.25 | A0A1S4FHA0;Q171G0;Q1DH07 |
|  | Citrate hydro-lyase | -1.82 | -0.86 | -2.07 | -1.05 | Q16ZG5;Q6SYX7;A0A4V1FMT3 |
|  | Transferrin | -1.79 | -0.84 | -1.85 | -0.89 | Q16NJ0;A0A6I8TLH7;Q16NI9;B6DRB0;A0A6I8TE78 |
|  | Titin | -9.21 | -3.20 | -3.70 | -1.89 | A0A6I8T6M3;A0A6I8T741;A0A1S4F231;A0A6I8T6S6 |

| Colour code |  |
| --- | --- |
|  | Significantly expressed (Log2Fold change= <-3.0) |
|  | Significantly expressed (Log2Fold change= <-2.0, >-3.0) |
|  | Significantly expressed (Log2Fold change= <-1.5, >-2.0) |
|  | Not significantly expressed (Log2Fold change= <-1.0, >-1.5) |
|  | Not significantly expressed (Log2Fold change= <-0.5, >-1.0) |
|  | Not significantly expressed (Log2Fold change= <-0.5) |
