# Supplementary material for: Proteomic analysis unveils host-parasite interactions in Aedes togoi infected with Dirofilaria immitis and Brugia pahangi
Source: PLoS One. 2025 Jul 9;20(7):e0326693. doi: 10.1371/journal.pone.0326693 (PMC12240324; doi:10.1371/journal.pone.0326693)
Supplement: S3 Table — (DOCX) [file pone.0326693.s003.docx]

**Table S3.** **Inconsistent upregulated and downregulated proteins of BPH and DIM compared to control**

| **No** | **Protein** | **BPH** | | **DIM** | | **Protein IDs** |
| --- | --- | --- | --- | --- | --- | --- |
|  |  | **Fold change** | **log2Fold change** | **Fold change** | **log2Fold change** |  |
|  | Phosphoglucomutase 1 | 1.39 | 0.47 | -1.48 | -0.56 | Q58I84;Q16U43 |
|  | Phosphoglycerate kinase | -2.10 | -1.07 | 1.48 | 0.57 | Q95UR6;Q8WQL0;Q8WQK9;Q95UR5;Q8WQK8;Q8WQL1;B1A651 |
|  | Pyruvate kinase | -2.32 | -1.21 | 1.45 | 0.54 | Q16LP4;Q16LP5;Q16F38 |
|  | 60S ribosomal protein L11 | -1.00 | -0.01 | 1.30 | 0.38 | Q16KL2;Q1HRK1 |
|  | Aconitate hydratase, mitochondrial | -1.19 | -0.25 | 1.27 | 0.35 | Q16KR4;Q17EL3 |
|  | Glucose-6-phosphate isomerase | -1.68 | -0.75 | 4.75 | 2.25 | Q16LE8;Q16KI0 |
|  | Proton-translocating NAD(P)(+) transhydrogenase | -1.45 | -0.54 | 1.17 | 0.22 | Q16LL0;A0A1S4FX45 |
|  | Succinate--CoA ligase ADP-forming subunit beta, mitochondrial | -1.02 | -0.03 | 5.30 | 2.41 | Q16P83;Q16P82 |
|  | Delta-1-pyrroline-5-carboxylate synthase | -1.24 | -0.31 | 3.36 | 1.75 | Q174N2 |
|  | Glutamate dehydrogenase (NAD(P)(+)) | -1.14 | -0.19 | 1.28 | 0.35 | Q16SW1 |
|  | Protein yellow | -1.26 | -0.33 | 1.96 | 0.97 | Q174P5;A0A1S4FEX1;Q173X2;A0A1S4FFE1 |
|  | AAEL010097-PA | -1.39 | -0.48 | 3.27 | 1.71 | Q16TW3 |
|  | 26S protease (S4) regulatory subunit, putative | -1.92 | -0.94 | 1.59 | 0.67 | Q16UJ3 |
|  | Laminin A chain, putative | -1.25 | -0.32 | 1.12 | 0.16 | Q16XT3;A0A1S4FKJ6 |
|  | Serine Protease Inhibitor (serpin) likely cleavage at SS | -1.11 | -0.14 | 1.05 | 0.07 | Q16Z02;A0A6I8TFQ0 |
|  | Syntaxin binding protein-1,2,3 | -1.10 | -0.14 | 2.01 | 1.01 | Q172R7;Q172R6 |
|  | Calcium-transporting ATPase | -2.28 | -1.19 | 2.54 | 1.34 | Q175R4;A0A6I8TD93;Q175R5;A0A1S4FE53;Q175R3 |
|  | Trehalose-6-phosphate synthase | -1.11 | -0.15 | 2.74 | 1.45 | Q176D3 |
|  | Vitellogenin-B | -4.17 | -2.06 | 8.97 | 3.17 | Q177I2;Q6U1K9 |
|  | Proteasome subunit alpha type | -4.96 | -2.31 | 1.69 | 0.76 | Q17EA3 |
|  | Ras-related protein Rab-7 | -1.31 | -0.39 | 1.01 | 0.01 | Q17F70 |
|  | Triosephosphate isomerase | -1.10 | -0.13 | 3.71 | 1.89 | Q17HW3 |
|  | Nipsnap | -1.10 | -0.14 | 2.19 | 1.13 | Q17Q08;A0A1S4EV42 |
|  | Protein kinase A cAMP-dependent catalytic subunit | -1.87 | -0.90 | 1.04 | 0.06 | Q1HQW4 |
|  | Calmodulin | -1.09 | -0.12 | 1.08 | 0.11 | Q1HQX3 |
|  | NADPH--cytochrome P450 reductase | -1.77 | -0.83 | 1.44 | 0.53 | Q17FM7;A0A6I8TSY4;Q16FP0;A0A6I8U8C1 |
|  | Putative secreted salivary protein | -1.72 | -0.78 | 1.10 | 0.14 | Q1HRT2;Q16HV0;Q16U86;Q16HV9;A0A1S4G0N6 |
|  | ADP-ATP translocase | -1.22 | -0.29 | 1.29 | 0.37 | Q1HRU0 |
|  | 40S ribosomal protein S3 | -1.69 | -0.76 | 4.23 | 2.08 | Q4F6X0;J9HFW1 |
|  | Proline dehydrogenase (Fragment) | -2.52 | -1.34 | 1.75 | 0.81 | A0A6I8TNR3;A0A1S4FZI4;A0A6I8TNM0;A0A6I8TFG7;Q16J67 |

**Table S3. Continued…**

| **No** | **Protein** | **BPH** | | **DIM** | | **Protein IDs** |
| --- | --- | --- | --- | --- | --- | --- |
|  |  | **Fold change** | **log2Fold change** | **Fold change** | **log2Fold change** |  |
|  | Muscle-specific actin 3 | 5.07 | 2.34 | -1.71 | -0.78 | Q17C87;Q1HRN5;Q6ELZ6;D9YSS9 |
|  | ATP synthase subunit D, mitochondrial | 1.67 | 0.74 | -1.00 | -0.01 | Q1HR21 |
|  | Delta-1-pyrroline-5-carboxylate synthase | -1.24 | -0.31 | 3.36 | 1.75 | Q174N2;Q2KQ99 |
|  | Protein-serinethreonine phosphatase | -1.09 | -0.13 | 1.39 | 0.48 | Q176D4;A0A6I8TCP2;A0A6I8TD03;A0A6I8T913;A0A6I8TDZ8 |
|  | Oxoglutarate dehydrogenase (succinyl-transferring) | -1.15 | -0.21 | 1.38 | 0.46 | A0A6I8TD30;Q175A4;A0A6I8T9A7;A0A6I8TDG4;Q175A3 |
|  | Glucose-6-phosphate isomerase | -1.68 | -0.75 | 4.75 | 2.25 | Q16LE8;Q16KI0 |
