# Supplementary material for: Proteomic analysis unveils host-parasite interactions in Aedes togoi infected with Dirofilaria immitis and Brugia pahangi
Source: PLoS One. 2025 Jul 9;20(7):e0326693. doi: 10.1371/journal.pone.0326693 (PMC12240324; doi:10.1371/journal.pone.0326693)
Supplement: S4 Table — (DOCX) [file pone.0326693.s004.docx]

**Table S4.** **Proteins exclusively identified BPH and DIM**

| **No** | **Protein** | **BPH** | **DIM** | **Protein IDs** |
| --- | --- | --- | --- | --- |
|  |  | **Intensity** | **Intensity** |  |
|  | Angiotensin-converting enzyme | 32416000 | 52534000 | J9HF68;A0A1S4G6D0;J9E9A1 |
|  | Beta-hexosaminidase | 14845000 | 15838000 | J9HI66;Q17BL1 |
|  | ACT complex subunit SSRP1 | 5797700 | 2490100 | Q0IEB2 |
|  | AAEL006431-PA | 8735600 | 13743000 | Q0IFA3 |
|  | Mitochondrial carrier | 17139000 | 19659000 | Q16ES8;A0A1S4G3V8 |
|  | Coatomer subunit alpha | 24469000 | 31615000 | Q16EV4;Q16K68;A0A1S4FYI5;A2I873 |
|  | AAEL013676-PA | 21168000 | 52880000 | Q16FL0 |
|  | AAEL013535-PA | 6846200 | 171930000 | Q16IU7 |
|  | Serine/threonine-protein phosphatase | 19741000 | 33419000 | Q16K96 |
|  | Importin subunit alpha | 60401000 | 56254000 | Q16KJ6 |
|  | 26S proteasome regulatory subunit 7 | 13359000 | 48186000 | Q16KL0 |
|  | CCT-theta | 33196000 | 82976000 | Q16L72;A0A1S4FXE3 |
|  | DNA replication licensing factor MCM6 | 12000000 | 18241000 | Q16LS4 |
|  | Vesicle-fusing ATPase (Fragment) | 9092600 | 35147000 | Q16MA3;Q16SH1 |
|  | AAEL012312-PA | 36807000 | 75194000 | Q16MG5 |
|  | 26S proteasome regulatory subunit S3 | 42266000 | 69368000 | Q16N14 |
|  | AAEL011870-PA | 17725000 | 45288000 | Q16NS4;A0A6I8TLC6 |
|  | 60S ribosomal protein L27 | 17554000 | 11734000 | Q16PM4;Q16FB1 |
|  | DNA replication licensing factor MCM3 | 28478000 | 76879000 | Q16NY8;A0A1S4FUM8 |
|  | AAEL011320-PA | 11123000 | 22643000 | Q16QE3 |
|  | Succinyl-CoA:3-ketoacid-coenzyme A transferase | 6626800 | 15112000 | Q16QY3;A0A6I8TVL2;A0A6I8TWN5 |
|  | Truncated ER mannose-binding lectin (Fragment) | 14215000 | 23047000 | Q16RM8;Q1HQV3;A0A0N8ES37 |
|  | AAEL010248-PA | 41145000 | 38504000 | Q16TF1 |
|  | SAM domain-containing protein | 7072500 | 11802000 | Q16TR2;A0A6I8TJ19;A0A0P6K0N0 |
|  | Isocitrate dehydrogenase NAD subunit, mitochondrial | 43350000 | 61910000 | Q16TS5;A0A1S4FPD7 |
|  | T-complex protein 1 subunit gamma | 75872000 | 227930000 | Q16U15 |
|  | Proteasome subunit alpha type | 23514000 | 33917000 | Q16UH5 |
|  | AAEL009882-PA | 11839000 | 19924000 | Q16UI5 |
|  | Neprilysin | 10005000 | 10054000 | Q16UJ2;A0A6R8GJR6;A0A6I8TIM5 |
|  | CCT-alpha | 40194000 | 82451000 | Q16W74;A0A1S4FLY4 |
|  | Complex I-B17 | 17480000 | 10809000 | Q16WX6 |
|  | Presequence protease, mitochondrial | 60106000 | 72199000 | Q16XJ0;A0A1S4FKL4 |
|  | Cdk1 | 24249000 | 48804000 | Q16Y81 |
|  | SURF1-like protein (Fragment) | 12647000 | 14560000 | Q16ZD9;Q16QM3;A0A6I8U5G9 |
|  | Fumarate hydratase | 28746000 | 71027000 | Q16ZK9;A0A6I8TFZ7;A0A6I8TFH2;Q16ZL0 |
|  | Bleomycin hydrolase | 12251000 | 23578000 | Q16ZW2;Q1HQS7;A0A6I8TFN9 |
|  | T-complex protein 1 subunit delta | 32371000 | 36552000 | Q171B7 |
|  | AAEL007297-PA | 26030000 | 49774000 | Q172T1 |
|  | DNA replication licensing factor MCM2 | 10614000 | 24329000 | Q173T8;A0A1S4FFD5 |
|  | Lon protease homolog, mitochondrial | 7213400 | 10723000 | Q176B8;A0A6I8T919 |
|  | AAEL006242-PA | 29832000 | 33894000 | Q176W5;A0A6I8U5P4 |
|  | Complex I-B15 | 32881000 | 46474000 | Q17H03 |
|  | Galactokinase | 5745000 | 6178200 | Q17H63;Q17H64;A0A6I8T786 |
|  | AAEL002493-PA | 13921000 | 15526000 | Q17I51 |
|  | Troponin t, invertebrate | 44402000 | 29136000 | Q17I94;A0A6I8T6J1;A0A6I8T6D9;Q17I95;A0A6I8T582;Q17I96 |
|  | Ras-related protein R-Ras2 | 33104000 | 41612000 | Q17J62;Q5QC98 |
|  | CCT-epsilon | 24704000 | 52735000 | Q17KD5;A0A1S4EZR6 |
|  | Coronin | 12479000 | 39260000 | Q17LF3;Q17LF4 |
|  | DNA replication licensing factor MCM7 | 22762000 | 38453000 | Q17ML5 |
|  | 26S protease regulatory subunit S10b | 23630000 | 38130000 | Q1HQM1 |
|  | RAB family GTPase | 13895000 | 25622000 | Q1HQR4 |
|  | T-complex protein 1 subunit eta | 78793000 | 111540000 | Q17P26;Q17P27 |
|  | MICOS complex subunit | 33887000 | 31810000 | Q1HQI8 |
|  | 60S ribosomal protein L36 | 40168000 | 38150000 | Q1HR17;Q17HK5 |
|  | 40S ribosomal protein S10 | 8704500 | 22611000 | Q1HR41 |
|  | Eukaryotic translation initiation factor 3 subunit F | 34277000 | 51684000 | Q1HR47 |
|  | 60S ribosomal protein L37 | 9029600 | 22602000 | Q1HR76 |
|  | 60S ribosomal protein L7 | 281290000 | 352950000 | Q1HR81 |
|  | 60S acidic ribosomal protein P0 | 26307000 | 21544000 | Q1HR99 |
|  | Glucose-1-phosphate uridylytransferase | 8941600 | 86454000 | A0A6I8TBP2;Q58I82;A0A1S4FB16 |
|  | 40S ribosomal protein S18 | 72102000 | 91801000 | Q1HRL8 |
|  | 40S ribosomal protein S23 | 7510000 | 54855000 | Q1HRM5 |
|  | 60S ribosomal protein L10 | 40250000 | 172950000 | Q1HRT6 |
|  | 40S ribosomal protein S5 | 44135000 | 56047000 | Q1HRT9 |
|  | Aspartate aminotransferase | 16483000 | 13535000 | Q5K6H3 |
|  | Ribonucleoside-diphosphate reductase | 3803300 | 45130000 | Q8WRS8;A0A1S4FQW9 |
|  | 60S ribosomal protein L23 | 17267000 | 17127000 | Q9GNE2 |
|  | Acetyl-CoA carboxylase | 4387400 | 50184000 | A0A6I8U7E0;A0A6I8U419;A0A6I8TTM8;A0A6I8U6N7;A0A6I8U908;Q176P0 |
|  | Glucose-1-phosphate uridylyltransferase | 56153000 | 86454000 | A0A6I8TBP2;Q58I82;A0A1S4FB16 |
