# Supplementary material for: Proteomic analysis unveils host-parasite interactions in Aedes togoi infected with Dirofilaria immitis and Brugia pahangi
Source: PLoS One. 2025 Jul 9;20(7):e0326693. doi: 10.1371/journal.pone.0326693 (PMC12240324; doi:10.1371/journal.pone.0326693)
Supplement: S5 Table — (DOCX) [file pone.0326693.s005.docx]

**Table S5.** **Proteins exclusively identified in DIM**

| **No** | **Protein** | **Intensity** | **Protein IDs** |
| --- | --- | --- | --- |
|  | Homogentisate 1,2-dioxygenase | 14051000 | Q16IJ1;A0A1S4FZN3 |
|  | Vitellogenic carboxypeptidase | 12681000 | P42660;A0A6I8TD36 |
|  | 4-hydroxyphenylpyruvate dioxygenase | 2980800 | Q16SY0;Q16FX9 |
|  | S-methyl-5-thioadenosine phosphorylase | 10900000 | Q16MW6 |
|  | AAEL007845-PA | 36604000 | Q170P3 |
|  | [AAEL012875-PA](https://www.uniprot.org/uniprot/A0A182GIM5) | 22557000 | Q1HQY2 |
|  | 60S ribosomal protein L30 | 47623000 | Q1HR35 |
|  | AAEL007777-PA | 10834000 | Q1HRK0 |
|  | AAEL010442-PA | 2980800 | Q16SY0 |
|  | Trehalose 6-phosphate phosphatase | 6926600 | Q16S69;A0A1S4FR40 |
