# Supplementary material for: Proteomic analysis unveils host-parasite interactions in Aedes togoi infected with Dirofilaria immitis and Brugia pahangi
Source: PLoS One. 2025 Jul 9;20(7):e0326693. doi: 10.1371/journal.pone.0326693 (PMC12240324; doi:10.1371/journal.pone.0326693)
Supplement: S6 Table — (DOCX) [file pone.0326693.s006.docx]

**Table S6.** **Proteins exclusively identified in BPH**

| **No** | **Protein** | **Intensity** |
| --- | --- | --- |
|  | Pyruvate dehydrogenase E1 component subunit alpha (Fragment) | 48673000 |
|  | AAEL004195-PA | 85795000 |
|  | Cytochrome c oxidase assembly factor 3 | 15700000 |
|  | AAEL001232-PA | 64483000 |
