# Supplementary material for: Proteomic analysis unveils host-parasite interactions in Aedes togoi infected with Dirofilaria immitis and Brugia pahangi
Source: PLoS One. 2025 Jul 9;20(7):e0326693. doi: 10.1371/journal.pone.0326693 (PMC12240324; doi:10.1371/journal.pone.0326693)
Supplement: S7 Table — (DOCX) [file pone.0326693.s007.docx]

**Table S7.** **Proteins exclusively identified in control**

| **No** | **Protein** | **Intensity** | **Protein IDs** |
| --- | --- | --- | --- |
|  | Calcium-transporting ATPase | 4592200 | A0A6I8TTA6;A0A6I8TSG8;A0A6I8TSG2;A0A6I8TTH7 |
|  | 40S ribosomal protein S16 | 6495600 | P62251 |
|  | AAEL015512-PA (Fragment) | 9268100 | Q1DGS2;Q1HR52 |
|  | AAEL007494-PA | 14279000 | Q171Z1 |
|  | AAEL003957-PA | 20015000 | Q1HQF5 |
|  | AAEL006014-PA | 21535000 | Q177U0;O96645;Q16E76 |
|  | AAEL003658-PA | 37527000 | Q17EW2;A0A6I8T892 |
|  | AAEL012905-PA | 76415000 | Q16KQ8;A0A6I8TVF9 |
|  | AAEL002759-PB | 133700000 | Q17H81;Q17H82;Q17H80 |
