# Supplementary material for: Proteomic analysis unveils host-parasite interactions in Aedes togoi infected with Dirofilaria immitis and Brugia pahangi
Source: PLoS One. 2025 Jul 9;20(7):e0326693. doi: 10.1371/journal.pone.0326693 (PMC12240324; doi:10.1371/journal.pone.0326693)
Supplement: S8 Table — (DOCX) [file pone.0326693.s008.docx]

**Table S8. Downregulated proteins of DIM compared to control**

| **No** | **Protein** | **DIM** | | **Protein IDs** |
| --- | --- | --- | --- | --- |
|  |  | **Fold change** | **log2Fold change** |  |
|  | Multifunctional fusion protein | -1.60 | -0.68 | Q17A27;Q16I13 |
