# Supplementary material for: Proteomic analysis unveils host-parasite interactions in Aedes togoi infected with Dirofilaria immitis and Brugia pahangi
Source: PLoS One. 2025 Jul 9;20(7):e0326693. doi: 10.1371/journal.pone.0326693 (PMC12240324; doi:10.1371/journal.pone.0326693)
Supplement: S9 Table — (DOCX) [file pone.0326693.s009.docx]

**Table S9. Upregulated and downregulated proteins of BPH compared to control**

| **No** | **Protein** | **BPH** | | **Protein IDs** |
| --- | --- | --- | --- | --- |
|  |  | **Fold change** | **log2Fold change** |  |
|  | AAEL008289-PA | 1.601 | 0.68 | Q16Z73 |
|  | NADH dehydrogenase [ubiquinone] 1 alpha subcomplex subunit 10, mitochondrial | 1.17 | 0.23 | Q1HQV6 |
|  | Laminin gamma 1 chain | 2.20 | 1.14 | Q17AS8; A0A6I8T7R7 |
|  | AAEL003110-PA | -2.04 | -1.03 | Q17GD7 |
|  | Alpha-galactosidase | -2.94 | -1.56 | Q17A03 |
